# Novel Stable Compounds in the C-H-O Ternary System at High Pressure

**DOI:** 10.1038/srep32486

**Published:** 2016-09-01

**Authors:** Gabriele Saleh, Artem R. Oganov

**Affiliations:** 1 Moscow Institute of Physics and Technology, 9 Institutsky Per., Dolgoprudny, Moscow Region, 141700, Russia; 2 Skolkovo Institute of Science and Technology, Skolkovo Innovation Center, 3 Nobel St., Moscow 143026, Russia; 3 Department of Geosciences and Department of Physics and Astronomy, Stony Brook University, Stony Brook, New York 11794-2100, USA; 4 International Center for Materials Discovery, Northwestern Polytechnical University, Xi’an, 710072, China

## Abstract

The chemistry of the elements is heavily altered by high pressure, with stabilization of many new and often unexpected compounds, the emergence of which can profoundly change models of planetary interiors, where high pressure reigns. The C-H-O system is one of the most important planet-forming systems, but its high-pressure chemistry is not well known. Here, using state-of-the-art variable-composition evolutionary searches combined with quantum-mechanical calculations, we explore the C-H-O system at pressures up to 400 GPa. Besides uncovering new stable polymorphs of high-pressure elements and known molecules, we predicted the formation of new compounds. A 2CH_4_:3H_2_ inclusion compound forms at low pressure and remains stable up to 215 GPa. Carbonic acid (H_2_CO_3_), highly unstable at ambient conditions, was predicted to form exothermically at mild pressure (about 1 GPa). As pressure rises, it polymerizes and, above 314 GPa, reacts with water to form orthocarbonic acid (H_4_CO_4_). This unexpected high-pressure chemistry is rationalized by analyzing charge density and electron localization function distributions, and implications for general chemistry and planetary science are also discussed.

The dramatic influence of high pressure (tens or hundreds of GPa) on reactivity is nowadays experimentally well established^1^. Crystal structure prediction approaches have become very effective in correctly anticipating experimental outcomes^2^,^3^. Among the numerous applications, these techniques have been widely exploited to predict high-pressure reactions^3^,^4^,^5^. Composition-pressure phase diagrams can be built by comparing the free energy of the most stable structures of elements and compounds at various pressures, so as to identify thermodynamically stable compositions. The latter are defined as those compounds for which any possible decomposition reaction is accompanied by a free energy (which at T = 0 K reduces to enthalpy) increase. In most cases, binary phase diagrams are targeted, for the large number of possible stoichiometries in a ternary system makes *ab initio* exploration computationally very demanding.

The C-H-O ternary phase diagram at high pressure is of paramount importance for planetary science. H_2_O and CH_4_, not necessarily in their intact molecular forms, are the major constituents of giant planets such as Neptune and Uranus, where pressure can reach hundreds of GPa^6^. Moreover, large icy satellites (*e.g.* Ganymede, Callisto and Titan)^7^ and comets^8^ all contain water ice, mixed with a number of volatiles such as CH_4_ and CO_2_, at pressures of up to a few GPa.

Some information on the C-H-O phase diagram is known. Generally, at ambient pressure, compounds other than water, carbon dioxide and methane are thermodynamically unstable (for hydrocarbons, see ref. 9), *i.e.* have higher Gibbs free energy than an isochemical mixture of C, H, O, H_2_O, CO_2_, CH_4_. While H_2_O and CO_2_ are expected to survive even in the terapascal regime^10^,^11^, above 155 GPa methane was predicted to decompose into mixtures of hydrogen and heavier hydrocarbons, namely ethane, butane and polyethylene^12^. On further compression, all alkanes transform into diamond-hydrogen mixtures^12^,^13^. The low-pressure part of the C-H-O phase diagram is particularly intricate for it incorporates a wide variety of inclusion compounds. Above 4 GPa, CH_4_ and H_2_ combine in various proportions to form molecular co-crystals^14^. When water is exposed to gases at low temperature and moderate pressures (typically 6–15 MPa^15^), gas hydrates may form. The latter are widely known compounds made by a framework of hydrogen-bonded water molecules encapsulating guest molecules. Common guests within the C-H-O composition are oxygen, hydrogen, carbon dioxide and small hydrocarbons. All these inclusion compounds are known to decompose when pressure rises beyond certain limits, typically a few GPa. However, some exceptions were reported^16^, *e.g.* the H_2_O:2H_2_ complex was predicted to be stable “at least up to 120 GPa^17^”. Within inclusion compounds, host and guest retain their molecular identities and interact through weak van der Waals forces. To the best of our knowledge, no thermodynamically stable molecules formed solely by C, H and O altogether are known to date.

In this work, the C-H-O phase diagram is explored in the pressure range 10–400 GPa by means of the powerful variable-composition evolutionary algorithm USPEX^18^ coupled with periodic density-functional calculations. Thorough, unbiased searches were performed sampling all possible C-H-O compositions, and a total of more than 125000 structures, generated by the evolutionary method, were relaxed to the closest minimum-enthalpy configuration. Chemical bonding in the resulting compounds was investigated by analyzing the real-space distribution of their charge density, in the framework of the Quantum Theory of Atoms in Molecules (QTAIM^19^), and of their electron localization function (ELF^20^). According to QTAIM, chemical bonds are mirrored in the charge density distribution at special saddle points, called ‘bond critical points’ (bcps). The evaluation of certain scalar properties at bcps provides information about the bond type. ELF is a simple measure of electron localization. Maxima appear in regions associated with chemical bonds, lone pairs and atomic cores, and electronic populations can be assigned to these chemical entities by integrating charge density within the basins corresponding to those maxima.

A schematic representation of the C-H-O pressure-composition phase diagram is reported in Fig. 1a,b. Our calculations recovered all the stable compounds known from previous crystal structure prediction works^10^,^11^,^12^,^17^,^21^,^22^,^23^. The crystal structures we obtained were either the same as those previously reported, or structurally and enthalpically (typically within 1 meV/atom) similar. We briefly summarize these findings (for more details, see Supplementary Section S2). In both H_2_O:H_2_ and H_2_O:2H_2_ hydrates, the water framework is based on the structure of ice Ic^17^. For these two compounds, our structures differ from those of ref. 17 only by the orientation of H_2_ guest molecules or by a donor-acceptor switch in the O-H∙∙∙O interaction. It is noteworthy that above 100 GPa, our H_2_O:2H_2_ crystal structure (Fig. 2a) and the reported one have different H_2_ orientation but their enthalpy is nearly indistinguishable (ΔE ≤ 0.5 meV/atom), suggesting that the guest molecules are rotationally disordered even at high pressure. For this compound, we determined for the first time its decomposition pressure: 153 GPa. We discovered a structure of ethane (Fig. 2e) that is more enthalpically favorable than the previously reported one. This reduces the previous estimate^12^ of the pressure of the decomposition reaction 2CH_4_→ C_2_H_6_+ H_2_ from 200 GPa down to 121 GPa. For water ice above 200 GPa, we obtained the *Pbcm* structure already proposed in previous *ab initio* studies^24^,^25^ (Fig. 2d). Concerning oxygen, our high-pressure calculations uncovered a previously unknown hexagonal structure reported in Fig. 2b (*P6*_*3*_*/mmc* space group), stable above 375 GPa. Similarly to its lower-pressure ξ-phase (space group *C2/m*)^21^, this *P6*_*3*_*/mmc* oxygen is a metallic molecular crystal (Supplementary Fig. S2). In the lower-pressure ε- and ξ-phases, the intermolecular distances within the *ab* crystallographic plane have different values, ε-phase even having exotic (O_2_)_4_ clusters^21^. In the *P6*_*3*_*/mmc* structure, instead, they become equal thereby conferring to the crystal its hexagonal symmetry.

New compounds were predicted to form. A 2CH_4_:3H_2_ (Fig. 2c) clathrate was found to be stable from <10 GPa up to 215 GPa. It has the same crystal structure throughout its pressure range of stability. Curiously, the topology of the host framework (Supplementary Fig. S13) is reminiscent of that of gas hydrates. Concerning the possible formation of molecules containing C, H and O, our results indicate that carbonic acid becomes thermodynamically stable above 0.95 GPa (effects of zero-point energy correction and temperature are discussed below). This was quite unexpected as this molecule is highly unstable at ambient conditions^26^. Its synthesis requires the use of high-energy radiation or strong acids and the resulting compound can only be isolated under high vacuum or in argon matrix and at very low temperatures^27^,^28^. Indeed, the decomposition of carbonic acid into water and carbon dioxide is highly exothermic, although in the absence of water it is hampered by a high kinetic barrier^29^. The low-pressure structure (space group *Pnma*, Fig. 3a) is composed of chains made by hydrogen-bonded molecules. The latter adopt an almost flat conformation in which hydrogens are in *cis* position with respect to the C=O bond. This type of HBs arrangement was shown to be the most energetically favorable at ambient conditions, ‘cis-cis’ being the most stable conformation of the isolated molecule^28^. The crystal structure of carbonic acid is significantly denser than water ice (2.147 *vs* 1.560 g/cm^3^ at 1 GPa). This fact has important implications for planetary science (see below). As pressure rises, carbonic acid polymerizes (p > 44 GPa), forming the structure shown in Fig. 3b. The -CO- backbone of each polymer is parallel to the *c* crystallographic axis, while hydroxyl groups form HBs joining the adjacent polymeric chains along the *b* direction. This type of structure is stable up to the highest investigated pressure (400 GPa), although the HB network rearranges above 240 GPa, leaving the CO backbone and the crystallographic space group unchanged. In the higher-pressure conformation (Fig. 3d), each polymer is hydrogen-bonded to the nearest neighbors along the two *ab* diagonals. This implies that, differently from the lower-pressure *Cmc2*_*1*_ structure, at high pressure the HB network joins all the polymers together. Finally, at 314 GPa, we detected an exothermic reaction between carbonic acid and water, leading to the formation of orthocarbonic acid (H_4_CO_4_, Fig. 3e). Besides being thermodynamically stable, the newly discovered compounds were ascertained to be dynamically stable by the absence of imaginary frequencies in their phonon dispersion curves (Supplementary Section S6).

The intricate chemistry described above can be rationalized by taking a closer look at the crystal structures and by analyzing the QTAIM properties and ELF distribution. First we note that, as molecular crystal *Pnma*-H_2_CO_3_ is compressed, the C-O hydroxyl bonds (C=O carbonyl bonds) shorten (elongate) and their bcps ellipticity increases (decreases), as shown in Supplementary Table S2. This indicates a progressive delocalization of the π orbital over the two formally single C-O1 bonds, eventually making them shorter than the formally double C=O2 bond. This behavior can be explained with the chemical scheme of Fig. 3c. As pressure rises, the weight of the resonance forms II and III increases. ELF distribution supports this hypothesis: a fourth maximum appears around O2 above 40 GPa (Fig. 4c–f), indicating a progressive shift from sp^2^ to sp^3^ hybridization, and at 100 GPa 5.8 out of 7.6 valence electrons are contained in the lone pairs basins (Supplementary Table S3). This trend can be seen as a pressure-induced destabilization of the double bond, which is likely to be an important factor contributing to the polymerization of carbonic acid. The latter is indeed associated with the breaking of the π bond and the concurrent formation of a new C-O σ bond, in a way which is reminiscent of the high-pressure behavior of CO_2_^10^. Accordingly, upon the *Pnma*→*Cmc2*_*1*_ phase transition, C-O bonds elongate and their ellipticity at bcp decreases (Table 1). HBs undergo important changes, too. Differently from the *Pnma* phase, in the *Cmc2*_*1*_ structure O-H and H···O interactions display similar bond lengths, degree of covalency (as measured by the kinetic energy density per electron^30^) and charge density at bcp (Table 1). Moreover, in passing from *Pnma* to *Cmc2*_*1*_ phase, a significant decrease (increase) in the population of the ELF basins corresponding to acceptor lone pairs (O-H bonds) takes place. Such a charge transfer from the acceptor lone pair to the donor-H fragment generally occurs in passing from weak to strong HBs^31^. Thus, along the *Pnma*→*Cmc2*_*1*_ phase transition, HBs strengthen and become noticeably more symmetric. The symmetrization of HBs increases as pressure rises, and in the *Cmc2*_*1*_ phase at 400 GPa O-H and H···O interactions are nearly indistinguishable (Table 1; in the following, these types of HBs will be referred to as ‘O-H-O bonds’, to distinguish them from weaker HBs). The different nature of HBs in the two phases of carbonic acid is even more evident in the ELF distribution (Fig. 4a,b), which for *Cmc2*_*1*_ is more akin to that of symmetric O-H-O bonds observed in the high-pressure phases of ice (ice X and *Pbcm*-ice, Supplementary Fig. S5). Noteworthy is the appearance of an ELF maximum on H atoms for all the investigated high-pressure HBs, a feature observed at ambient pressure only in 3c-4e bonds such as those of F-H-F^−^and H_2_O-H-OH_2_^+^ compounds^32^.

The reaction H_2_CO_3_ + H_2_O → H_4_CO_4_ becomes exothermic at high pressure due to the volume reduction (*i.e.* is driven by the pV term in the free energy) and is homodesmic: the total number of C-O and O-H-O bonds remains constant, and so does the total number of oxygen’s lone pairs not involved in O-H-O bonds. These lone pairs form weaker HBs with the H atoms of O-H-O bonds in carbonic and orthocarbonic acids (Fig. 5). Such HBs are not present in water, hence their number increases in passing from the reactant mixture to H_4_CO_4_. The formation of additional interactions is expected to be associated with a more efficient packing; in fact, by comparing the ELF basins of orthocarbonic acid with those of the reactants (Fig. 6), it emerges that the O-H-O bonds of water significantly shrink along the formation reaction, thereby overcompensating the volume increase observed for other basins. These results suggest that the occurrence of additional HBs upon the formation of orthocarbonic acid plays a fundamental role in the achievement of the volume reduction responsible for its high-pressure stability. We observed similar results (formation of O···H contacts and ELF trends) in a metastable polymorph of H_4_CO_4_ (Supplementary Section S8), which is structurally different from the structure discussed above, but is also enthalpically favorable over the H_2_O + H_2_CO_3_ mixture above 395 GPa.

We now move to discuss the implications of our results for general chemistry and planetary science. The long-standing view that inclusion compounds systematically decompose at low pressures of a few GPa has been refuted in light of a number of counter-examples discovered during the last decades. However, only hydrates were known to persist above 50 GPa^16^. Our 2CH_4_:3H_2_ compound not only sets a new upper limit for the stability of inclusion compounds, but also introduces a qualitative shift of views, for it broadens the classes of inclusion compounds stable at very high pressures. In fact, several nCH_4_:mH_2_ crystals were experimentally found to be stable up to 8 GPa, and CH_4_:2H_2_ was compressed up to 30 GPa without showing any sign of decomposition^14^. The difference in the stoichiometry between such compound and the one presented here might be traced back to a number of causes, for example the possible metastability of the experimentally detected phase above 10 GPa. Remarkably, the existence of 2CH_4_:3H_2_ affects the high-pressure chemistry of methane: i) it lowers the decomposition pressure of pure methane crystals down to 93 GPa, much lower than previous estimates^12^ (Fig. 1a), and ii) allows methane molecules to survive up to 215 GPa (Fig. 1b), *i.e.* at a higher pressure higher than previously reported.

Concerning carbonic acid, its discovered stability already at moderate pressure opens new important possibilities for synthesizing and stabilizing this elusive molecule. However, it must be pointed out that direct comparison between the calculated and experimental formation pressures is complicated by two problems: the influence of lattice vibrations (temperature effects and zero-point energy correction) and the approximations made within the DFT approach. We have tackled these two issues by evaluating how the formation pressure of H_2_CO_3_ is affected by phonons (within the harmonic approximation) and by the use of different computational settings (including changes in the basis set and exchange-correlation functional). The pressure required for stabilizing carbonic acid shifts to 1.45 GPa when zero-point vibrational energy is included, and then weakly increases with temperature (Fig. 1c,d). All the tested computational approaches reproduced the pressure-induced stabilization of carbonic acid, although the formation pressure showed some variations (Supplementary Table S4). Overall, a realistic estimate of the formation pressure of carbonic acid would be the range 0.6–1.6 (0.75–1.75) GPa at 100 (300) K. Similar pressures occur on the bed of water oceans of icy satellites^7^. There, both water ice and carbon dioxide are present, hence carbonic acid is likely to form. Moreover, its high density implies that, once formed, carbonic acid will sink to the bottom of ice layers, just above the rocky cores, thereby experiencing an even greater pressure. In such a scenario, carbonic acid insulates water ice from the core. This would modify the chemical composition models for these celestial bodies, which now include possible reactions between water and rocky compounds such as ferromagnesiansilicates^7^.

Very recently (in fact, while this paper was being finalized) Wang *et al.*^33^ detected the formation of carbonic acid in compressed H_2_O:CO_2_ mixtures, which confirms our prediction. The authors hypothesized carbonic acid to be a metastable product of a complex, non-equilibrium process, while our findings lay down a theoretical ground for this discovery and assert carbonic acid as a thermodynamically stable compound under pressure. Our calculated IR and Raman spectra agree well with the measured ones (Supplementary Section S9, which contains a detailed comparison between experimental findings from literature and our theoretical results). This indicates that the H_2_CO_3_ crystal structure presented here is either the observed one, which is still not determined experimentally, or one with a very similar crystal packing. In fact, the energy landscape of H_2_CO_3_ is very complex and features many low-lying minima with comparable energies^34^, hence a systematic exploration of all of them is needed in order to reliably determine the correct structure. Such extensive analysis will be the subject of our future research.

In conclusion, we have carried out a thorough DFT investigation of the C-H-O phase diagram up to 400 GPa. For each composition, the most stable structures at various pressures were obtained by the powerful evolutionary algorithm USPEX. Our calculations predicted the formation of several new compounds. An inclusion compound, 2CH_4_:3H_2_, was found to be stable up to the unprecedented pressure of 215 GPa. Carbonic acid was predicted to be stable above 1 GPa, remaining stable throughout the investigated pressure range and polymerizing above 44 GPa. At 314 GPa it reacts with water to form orthocarbonic acid, H_4_CO_4_. An extensive chemical bonding analysis was performed, which provided important insights such as the pressure-induced stabilization of resonance forms of molecular carbonic acid and the role played by HBs in the stabilization of orthocarbonic acid. This novel chemistry should have major implications for planetary science.

## Methods

The variable-composition evolutionary approach implemented in USPEX, scans the structural and chemical spaces and seeks the thermodynamically stable compounds. The first generation of structures was mostly produced randomly (stable structures obtained from previous runs were also added). The subsequent generations were produced by both symmetric random generator and by applying variation operators (described in ref. 35) to the most promising structures (65% of the current generation are allowed to produce the next generation). Details of all USPEX runs can be found in Supplementary Table S1. Exhaustive description of the method^35^,^36^ and studies where the approach reliability was confirmed by comparison with experiments^3^,^12^,^21^,^37^ are reported elsewhere. The VASP code^38^ was used for structure relaxations and enthalpy calculations. We adopted the Perdew-Burke-Ernzerhof (PBE) functional^39^ in the framework of the all-electron projector augmented wave (PAW) method^40^, with ‘hard’ PAW potentials, plane wave kinetic energy cutoff of 850 eV and a uniform, Γ-centered grid with 2π*0.056 Å^−1^ spacing for reciprocal space sampling. In the 0–10 GPa range, where dispersion interactions play an important role, we employed for VASP calculations a van der Waals functional (optB88-vdw^41^). At these pressures, a slightly looser reciprocal space sampling of 2π*0.064 Å^−1^ was adopted. Note that at each pressure the stability of each compound was ascertained by considering the most stable form of the reactants, including structures from literature. To perform the chemical bonding analysis, we carried out single-point calculations (at the geometry obtained from VASP) with the CRYSTAL14 code^42^. Within the latter, crystal orbitals are described in terms of atom-centered functions. The employed basis set was of ‘triple-ξ plus polarization’ quality, whose functions were optimized for solid-state calculations^43^. In order to better describe intermolecular interactions, we augmented the basis set with d orbitals on H atoms taken from ref. 44. To make the basis set apt for high-pressure calculations, the exponents of the radial part of the outermost functions of each shell were contracted by a factor 1.44 and 1.69 for calculations below and above 200 GPa, respectively. The bond critical point analysis and the determination of atomic basins within the QTAIM framework was done using the TOPOND code, now implemented within CRYSTAL14. The latter was also exploited to evaluate grid files of scalar properties (such as charge density and ELF), subsequently converted in the standard ‘cube’ format using the NCImilano code^45^. The integration of quantities within ELF basins, not implemented in TOPOND, was performed with the critic2 code^46^, and in particular the grid-based Yu-Trinkle algorithm^47^ was exploited. The input grids were 400 × 400 × 400, spanning the whole unit cell. Phonon dispersion curves, and phonon contribution to the free energy of formation were calculated by means of the finite displacement method implemented in the PHONOPY code^48^. Further details concerning phonon calculations are reported in Supplementary Section S6. Images of structures and 2D maps/isosurfaces were produced with Diamond^49^ and VESTA^50^ software, respectively. Infrared and Raman spectra (reported in Supplementary Section S9) were calculated by means of the Coupled Perturbed Kohn-Sham approach implemented in CRYSTAL14^51^,^52^,^53^.

## Additional Information

**How to cite this article**: Saleh, G. and Oganov, A. R. Novel Stable Compounds in the C-H-O Ternary System at High Pressure. *Sci. Rep.* **6**, 32486; doi: 10.1038/srep32486 (2016).

## Supplementary Material

Supplementary Information

## Figures and Tables

**Figure 1:**
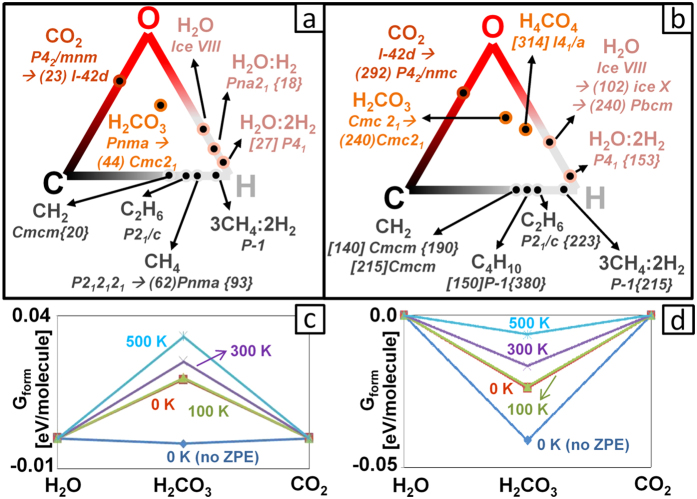
Summary of stable phases in the C-H-O phase diagram and convex hull for the CO_2_-H_2_O system. Stable phases in the C-H-O system at T = 0 K are reported for the ranges 10–100 GPa (**a**) and 100–400 GPa (**b**). For each compound, we report the space group(s) and the predicted phase transition pressures (in GPa), the latter in round brackets. Numbers in square (curly) brackets indicate the formation (decomposition) pressures. In (**a**), the formation pressure is not reported for those compounds already stable at 10 GPa. For elements, a detailed description of the known phases and the comparison with the ones obtained in our calculations is reported in Supplementary Section S2. A complete representation of the C-H-O phase diagram at various pressures, including Gibbs triangles, is reported in Supplementary Fig. S6. We also report the convex hulls at various temperatures for the H_2_O-CO_2_ system at 1 GPa (**c**) and 2 GPa (**d**). ‘no ZPE’ represents the value obtained neglecting the zero-point (vibrational) energy correction.

**Figure 2:**
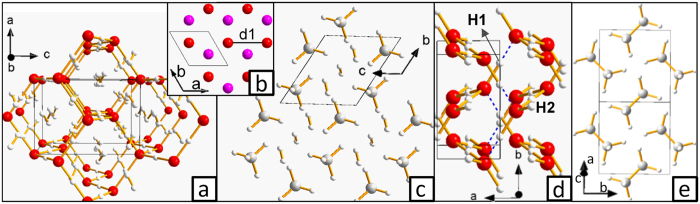
Novel crystal structures in the C-H-O phase diagram. (**a**) H_2_O:2H_2_, *I4*_*1*_*a* space group, at 100 GPa. (**b**) high pressure phase of oxygen, *P6*_*3*_*/mmc* space group, at 400 GPa. O_2_ molecules are oriented parallel to the *c* axis, with O atoms forming planes of triangular tiling parallel to the *ab* crystallographic plane (molecules at different heights are shown in red and violet). The distance between nearest neighbor molecules (d1 in the picture) is 1.878 Å at 400 GPa. This structure can be described as a hexagonal close packing, where instead of spheres there are O_2_ molecules. (**c**) 2CH_4_:3H_2_ inclusion compound, *P-1* space group, at 100 GPa. This structure is also reported in Supplementary Fig. S13, where the host framework topology is highlighted. (**d**) *Pbcm*-H_2_O at 400 GPa. There are 3 symmetry-independent atoms: 2 H (labeled in the picture) and 1 O. (**e**) ethane, space group *P2*_*1*_*/c*, at 100 GPa. In this and other pictures, C, H, and O atoms are colored in grey, white and red, respectively. Bonds (including symmetric O-H-O) are represented as yellow sticks while H···O interactions of HBs having OHO angle and H···O distance greater than 150° and shorter than 1.3 Å, respectively, are indicated as dotted blue lines.

**Figure 3:**
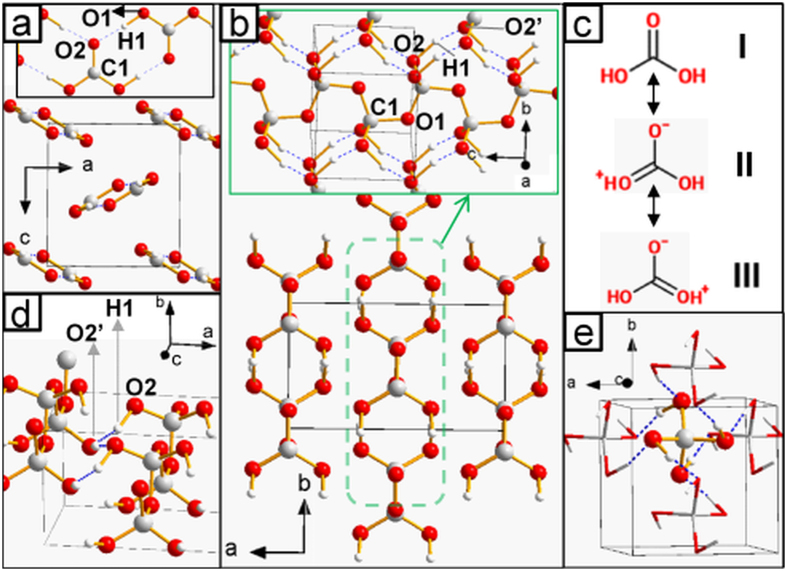
Crystal structures and chemical schemes for carbonic acid (H_2_CO_3_) and orthocarbonic acid (H_4_CO_4_). (**a**) *Pnma*-H_2_CO_3_ at 1 GPa. Chains of hydrogen-bonded molecules (displayed in the inset) extend along the *b* axis. The resonance forms discussed in the main text are shown in (**c**). (**b**) *Cmc2*_*1*_-H_2_CO_3_, low-pressure form (44–240 GPa) at 100 GPa. Polymers extend along the *c* axis, and they are shown in the inset. (**d**) hydrogen bonds in the high-pressure (above 240 GPa) form of *Cmc2*_*1*_-H_2_CO_3_ (**e**) I4_1_/a-H_4_CO_4_ at 400 GPa; we show a single molecule (represented in ‘ball-and-stick’ model), along with all its hydrogen-bonded neighbors (‘stick model’). We represent as dotted, blue lines those hydrogen bonds having OHO angle and H···O distance greater than 150° and shorter than 1.3 Å, respectively. For all the structures having more than one symmetry-independent atom of each type, the labels adopted in Figs 4,5 and 6 and Table 1 are shown.

**Figure 4:**
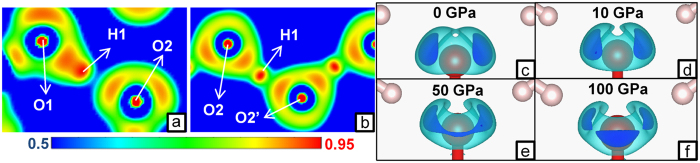
ELF distribution in carbonic acid. The ELF distribution at 100 GPa in the plane containing the O-H···O interaction is shown for *Pnma*-H_2_CO_3_ (**a**) and *Cmc2*_*1*_-H_2_CO_3_ (**b**). The color scale is reported below the pictures. (**c**–**f**) ELF isosurfaces showing the lone pairs of the O2 atom in *Pnma*-H_2_CO_3_ at various pressures (isosurface relative to C-O bond omitted for clarity). The adopted isovalues are 0.850 (light blue) and 0.875 (dark blue). Further 3D and 2D representations of ELF distribution in H_2_CO_3_, H_4_CO_4_ and H_2_O can be found in Supplementary Figs S4 and S5.

**Figure 5:**
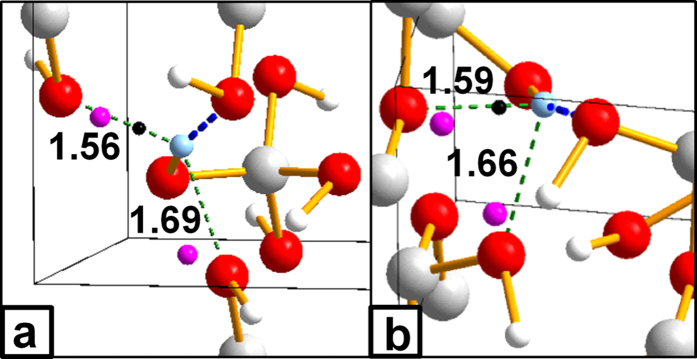
Hydrogen bonds in carbonic and orthocarbonic acids at 400 GPa. (**a**) orthocarbonic acid (**b**) carbonic acid. Each picture shows the coordination sphere of the (only) symmetry-independent hydrogen atom, colored in light blue. The short H···O contacts shown in Fig. 3 are represented by dotted, thick, blue lines. All other H···O contacts within 1.7 Å are indicated by dotted, thin, green lines, and their bond length (Å) is also reported. For each of the latter contacts, we also show the oxygen ELF maximum (violet sphere) closest to the hydrogen atom and the associated bond critical points (black, small sphere), where present (*i.e.* one H···O contact of each compound). The formation of additional H···O interactions is also mirrored in H atoms being drifted away from the line joining the two closest oxygens, the OHO angles being 151° (**a**) and 160° (**b**). For comparison, in water, where there are no available lone pairs to form additional HBs, the OHO angles are 179°–180°, no H-O bcps other than those of O-H-O bonds are found, and the shortest of the secondary H···O contacts measures 1.84 Å.

**Figure 6:**
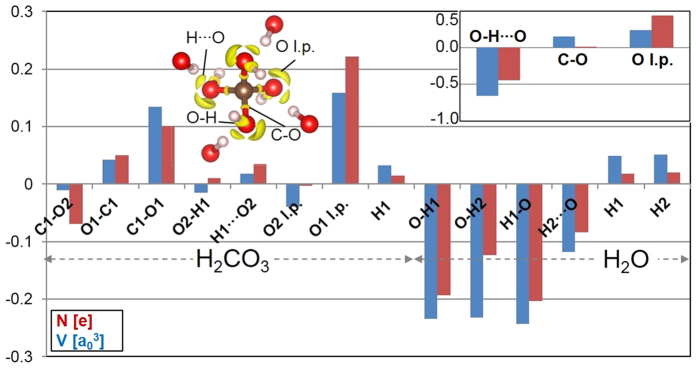
Variation of properties of valence ELF basins along the homodesmic reaction H_2_O + H_2_CO_3_ → H_4_CO_4_ at 400 GPa. As the number and types of ELF basins does not change along the reaction, a direct comparison of their properties between product and reactants can be made. Each bar in the histogram corresponds to a given bond/lone pair ELF basin of the reactants and indicates whether its volume and electron population (blue and red, respectively) increases or decreases in forming a basin of the same type in H_4_CO_4_, the latter having only one symmetry-independent basin of each type (C-O, O-H, H, H···O and O l.p.). For example, the value of the leftmost blue bar is obtained as V(C-O of H_4_CO_4_)-V(C1-O2 of H_2_CO_3_), V being the basin volume. For bonds, the same nomenclature as Table 1 is adopted, while “l.p.” refers to those oxygen’s lone pairs which are not involved in strong, (almost) symmetric HBs, the latter being referred to as O-H-O bonds in the text. A representative ELF isosurface plot of one H_4_CO_4_ molecule in the crystal is also shown (isovalue = 0.86), along with the O-H fragments of neighboring molecules which act as donor of strong HBs. The isosurfaces corresponding to each type of valence basin are labeled (except those on H positions, which are hidden inside the sphere representing the H atom). Inset in the upper right corner shows the total variation of various types of basins (O-H∙∙∙O is the sum of O-H, H, and H∙∙∙O basins) along the reaction. A complete list of volume and charge of valence ELF basins is reported in Supplementary Table S3.

**Table 1:** ELF and QTAIM properties of symmetry-independent bonds for selected compounds.

bond, A-B	d_AB_ [Å]	ρ_BCP_ [e/a_0_^3^]^a^	G_BCP_/ρ_BCP_[Eh/e]^b^	ELF pop.[n]^c^
Cmc2_1_-H_2_CO_3_(100GPa)				
O1-C1	1.363	0.31 {0.05}	0.79	1.48
C1-O1	1.336	0.32 {0.03}	0.90	1.52
C1-O2	1.325	0.34 {0.03}	0.85	1.57
O2-H1	1.134	0.22	0.51	1.61
H1∙∙∙O2	1.165	0.20	0.58	1.84
Pnma-H_2_CO_3_(100GPa)				
C1-O2	1.260	0.38 {0.11}	1.31	1.85
C1-O1	1.234	0.40 {0.16}	1.48	2.04
O1-H1	1.053	0.28	0.39	1.59
H1∙∙∙O2	1.261	0.15	0.69	1.98
Cmc2_1_-H_2_CO_3_(400GPa)				
O1-C1	1.286	0.36	1.23	1.53
C1-O1	1.277	0.37	1.26	1.58
C1-O2	1.236	0.41	1.39	1.70
O2-H1	1.063	0.27	0.65	1.66
H1∙∙∙O2^d^	1.066	0.27	0.66	1.63
I4_1_/a-H_4_CO_4_ (400GPa)				
C-O	1.268	0.38	1.24	1.63
O-H	1.058	0.28	0.61	1.67
H∙∙∙O^d^	1.096	0.25	0.69	1.66
Pbcm-H_2_O (400 GPa)				
O-H2	1.044	0.29	0.68	1.87
O-H1	1.033	0.30	0.66	1.80
H1∙∙∙O^d^	1.054	0.28	0.70	1.75

^a^Charge density at the bond critical point. For C-O bonds at 100 GPa, we report in curly brackets their ellipticity. The latter is defined as ε(*r*) = [λ_1_(*r*)/λ_2_(*r*)]-1, where λ_n_ is the n-th lowest eigenvalue of the charge density Hessian matrix. Ellipticity measures the deviation of charge density distribution along the internuclear axis from the cylindrical symmetry characteristic of σ bonds.

^b^kinetic energy density per electron.

^c^Electron population of the ELF basins joining the core ELF basins of atoms A and B (generally referred to as ‘disynaptic basins’). For H···O interactions, the reported value corresponds to the acceptor lone pair.

^d^These interactions are referred to as part of O-H-O bonds in the main text, due to the fact that these O-H···O bonds are very close to symmetrical HBs. Only those hydrogen bonds having OHO angle and H···O distance greater than 150° and shorter than 1.3 Å, respectively, are listed.
