# Supplementary material for: Novel Stable Compounds in the C-H-O Ternary System at High Pressure
Source: Sci Rep. 2016 Sep 1;6:32486. doi: 10.1038/srep32486 (PMC5007508; doi:10.1038/srep32486)
Supplement: Supplementary Information [file srep32486-s1.docx]

Novel Stable Compounds in the C-H-O Ternary System at High Pressure

Gabriele Saleh^a*^ , Artem R. Oganov^abcd^

a Moscow Institute of Physics and Technology, 9 Institutsky Per., Dolgoprudny, Moscow Region, 141700, Russia.

b Skolkovo Institute of Science and Technology, Skolkovo Innovation Center, 3 Nobel St., Moscow 143026, Russia.

c Department of Geosciences and Department of Physics and Astronomy, Stony Brook University, Stony Brook, New York 11794-2100, USA

d International Center for Materials Discovery, Northwestern Polytechnical University, Xi’an, 710072, China

*Correspondence and requests for materials should be addressed to G. S.

(email: gabrielesaleh@outlook.com)

SUPPLEMENTARY INFORMATION

Index

[Section S1. Details of USPEX calculations. 3](#_Toc457204818)

[Section S2. Detailed comparison between the structures obtained in this work and the previously reported ones. 3](#_Toc457204819)

[Section S3. Additional QTAIM and ELF results for H_2_CO_3_, H_4_CO_4_ and H_2_O. 7](#_Toc457204820)

[Section S4. C-H-O phase diagram and structural parameters of newly discovered crystal structures. 10](#_Toc457204821)

[Section S5. H_2_CO_3_ formation pressure calculated with different basis sets and DFT functionals. 15](#_Toc457204822)

[Section S6. Phonon dispersion curves of newly discovered compounds. 16](#_Toc457204823)

[Section S7. Additional representations of 2CH_4_:3H_2_ crystal structure 20](#_Toc457204824)

[Section S8. Structure and bonding of a polymorph of H_4_CO_4_ . 20](#_Toc457204825)

[Section S9. Comparison of calculated IR and Raman spectra of H_2_CO_3_ with previously reported experimental measurements. 22](#_Toc457204826)

[REFERENCES 24](#_Toc457204827)

# Section S1. Details of USPEX calculations.

**Table S1.**Details of each USPEX run carried out for the present study.

| calc. n | compositions  explored | pressure (GPa) | n. of structures in each generation | n. of generations | max number of atoms in the unit cell | total number of structures sampled |
| --- | --- | --- | --- | --- | --- | --- |
| 1^a^ | ternary,  C-H-O | 10 | 120 | 80 | 20 | 9600 |
| 2^a,b^ |  | 10 | 80 | 25 | 42 | 2000 |
| 3 |  | 50 | 90 | 55 | 20 | 4950 |
| 4 |  | 50 | 100 | 33 | 36 | 3300 |
| 5 |  | 100 | 120 | 58 | 20 | 6960 |
| 6 |  | 100 | 60 | 21 | 24 | 1260 |
| 7 |  | 150 | 90 | 65 | 20 | 5850 |
| 8 |  | 200 | 120 | 80 | 20 | 9600 |
| 9 |  | 200 | 120 | 92 | 20 | 11040 |
| 10 |  | 200 | 100 | 37 | 36 | 3700 |
| 11 |  | 250 | 80 | 65 | 32 | 5200 |
| 12 |  | 300 | 90 | 80 | 20 | 7200 |
| 13 |  | 300 | 120 | 80 | 20 | 9600 |
| 14^b^ |  | 300 | 80 | 47 | 42 | 3760 |
| 15^b^ |  | 300 | 80 | 99 | 42 | 7920 |
| 16 |  | 400 | 120 | 80 | 20 | 9600 |
| 17 |  | 400 | 120 | 80 | 20 | 9600 |
| 18 | binary,  CO_2_-H_2_O | 100 | 90 | 25 | 36 | 2250 |
| 19 |  | 200 | 100 | 30 | 36 | 3000 |
| 20 |  | 300 | 80 | 33 | 36 | 2640 |
| 21 |  | 400 | 100 | 30 | 36 | 3000 |
| 22 |  | 400 | 100 | 13 | 36 | 1300 |
| 23^a^ | fixed composition:  H_2_CO_3_ | 2 | 40 | 40 | 24 | 1600 |
| 24 | fixed composition: H_4_CO_4_ | 350 | 40 | 21 | 36 | 840 |

a)optB88-vdw functional^[[1]](#endnote-2)^ employed b) this calculations were performed as last, hence random structures were produced considering only reasonable stoichiometries (based on the experience gained from previous calculations).

# Section S2. Detailed comparison between the structures obtained in this work and the previously reported ones.

In this section, we discuss the relevant details concerning the structures we obtained from the USPEX runs listed in Table S1. We compare our structures with those reported in previous literature studies in the investigated pressure range (10-400 GPa).

Two comments are important here: first, the vast majority of these results were obtained in variable-composition calculations, which are much more challenging than traditional fixed-composition calculations. It is very encouraging that even in these challenging searches either correct ground states, or structures energetically nearly degenerate with ground-state ones, were found in most cases (the only exception being butane, for which a noticeably metastable, but chemically reasonable, structure was predicted). In a few cases, our searches discovered new ground states, not found before. Second, in all our analyses we used the most stable structures - found through comparison of the energetics of all previously published and currently found structures.

We found for ethane a structure more stable than the previously reported one, as discussed in the main text. For the following elements/compounds, we recovered the same structures as expected from previous studies: C (diamond throughout the 10-400 GPa range), O, CO_2_, H_2_O, CH_2_ (*Cmcm*), H_2_O:2H_2_ ,H_2_O:H_2_. Actually, for the latter we obtained a structure (*P2_1_2_1_2_1_* space group, Fig. S1) which differ from that of ref. ^[[2]](#endnote-3)^ by a donor-acceptor switch in the O-H∙∙∙O interaction. At 10 GPa, our structure is more stable by 0.04 meV/atom (optB88-vdw functional). For H_2_O:2H_2_, instead, our structure differs from the published one by the orientation of H_2_ molecules (see main text).


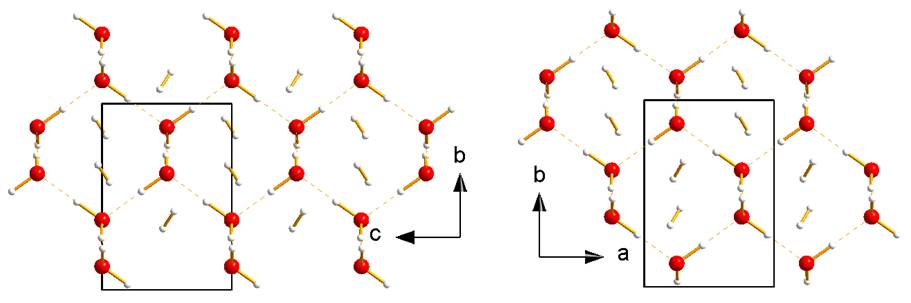


**Figure S1.** *P2_1_2_1_2_1_* structure of H_2_O:H_2_, viewed from two different orientations (hydrogen bonds indicated as dotted yellow lines).

For water, we obtained the sequence of structures expected from previous studies^[[3]](#endnote-4),^^[[4]](#endnote-5)^ *i.e.*: ice VIII-ice X- ice *Pbcm*. Disordered structures, *e.g.* the ones proposed to be an intermediate between ice VIII and ice X, could clearly not be detected in our static calculations. For the calculations carried out below 10 GPa to determine the formation pressure of H_2_CO_3_ , we also considered phases XV and II of ice. The transition pressures determined by our calculations below 10 GPa were 0.6 (ice II-ice XV) and 1.5 GPa (ice XV-ice VIII), in good agreement with experimental estimates^[[5]](#endnote-6)^.

The possible crystal structures of oxygen in the pressure range 10-250 GPa were thoroughly computationally explored in ref. ^[[6]](#endnote-7)^. The most stable structures obtained were *Cmcm*^[[7]](#footnote-2)^ and *C2/m*, below and above 40 GPa, respectively (the *C2/m* structure was proposed as a candidate for the ξ phase of oxygen). In the high-pressure ranges investigated in such work, a *C2/c* structure was found to be enthalpically competitive with the C2/m above 100 GPa. We obtained all these 3 structures, besides the *P6_3_/mmc* (most stable phase above 375 GPa) discussed in the main text. Below we report the density of states of *P6_3_/mmc*-O at 400 GPa, which shows its metallic character.


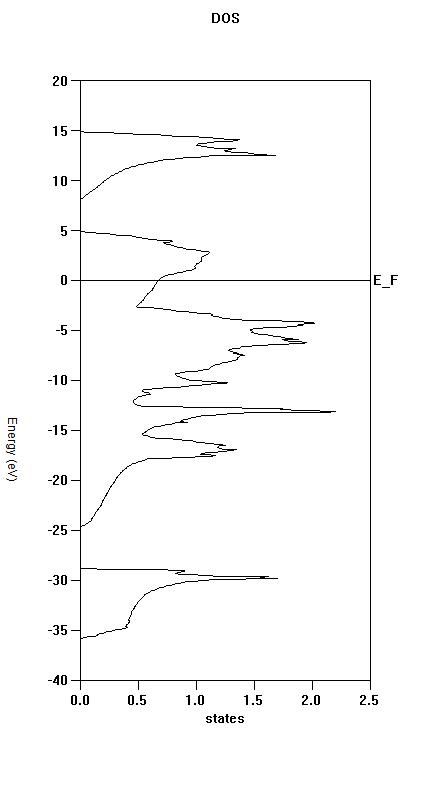


**Figure S2.** Density of states of *P6_3_/mmc* oxygen at 400 GPa. Vertical axis: E (eV). Horizontal axis: density of states (eV^-1^).

The phase diagram of CO_2_ was computationally investigated by Lu *et al.*^[[8]](#endnote-8)^. We obtained the expected sequence of structures in the 10-400 GPa range, namely *P4_2_/mnm*🡪 *I-42d* 🡪 *P4_2_/nmc*. At 10 GPa, the *P4_2_/nmn* structure is the most stable when optB88-vdw functional is adopted, as in our calculations, while in ref. 7 the (molecular) *Pa-3* phase was found to be the global minimum up to 10.3 GPa, due to the use of PBE functional. Note that the *Pa-3* 🡪 *P4_2_/mnm* phase transition is experimentally known^[[9]](#endnote-9)^ to take place below 11 GPa. However, an accurate determination of the low-temperature transition pressure was not reported.

Only for hydrogen, methane and butane our calculations did not recover all the most stable structures.

Concerning hydrogen, experiments detected 5 phases in the 1-400 GPa range^[[10]](#endnote-10)^, although only for the lowest pressure phase the crystal structure could be experimentally determined^[[11]](#endnote-11)^. At high pressure, many structures were found to lie in a narrow energy range, and some of them were proposed as possible candidates for the observed phases^[[12]](#endnote-12)^. Besides the experimental structure, we use the ones of ref. 11 as a benchmark for our results. In such work, a thorough DFT crystal structure prediction investigation in the range 100-400 GPa was carried out. We recovered some of the structures reported in ref. 11, besides the (experimental) low-pressure *Pa3* structure reported in ref. 10. For those pressure ranges in which we found structures different from the previously reported ones, the latter were generally less stable by a maximum of 0.8 meV/atom. The comparison among the enthalpy of the various structures is reported in Fig. S3.

The most stable structures of methane from 10 GPa up to its decomposition pressure are *P2_1_2_1_2_1_* (p< 63 GPa) and *Pnma* (P ≥ 63 GPa)^[[13]](#endnote-13)^. We obtained only the latter phase (besides many other less stable ones). The *P2_1_2_1_2_1_* vs *Pnma* enthalpy difference decreases with pressure. At 10 GPa, it is 0.57 meV/atom.

Butane was shown^12^ to have only one phase throughout its stability range. We obtained a less stable (up to 29 meV/atom) structure of *P-1* symmetry.


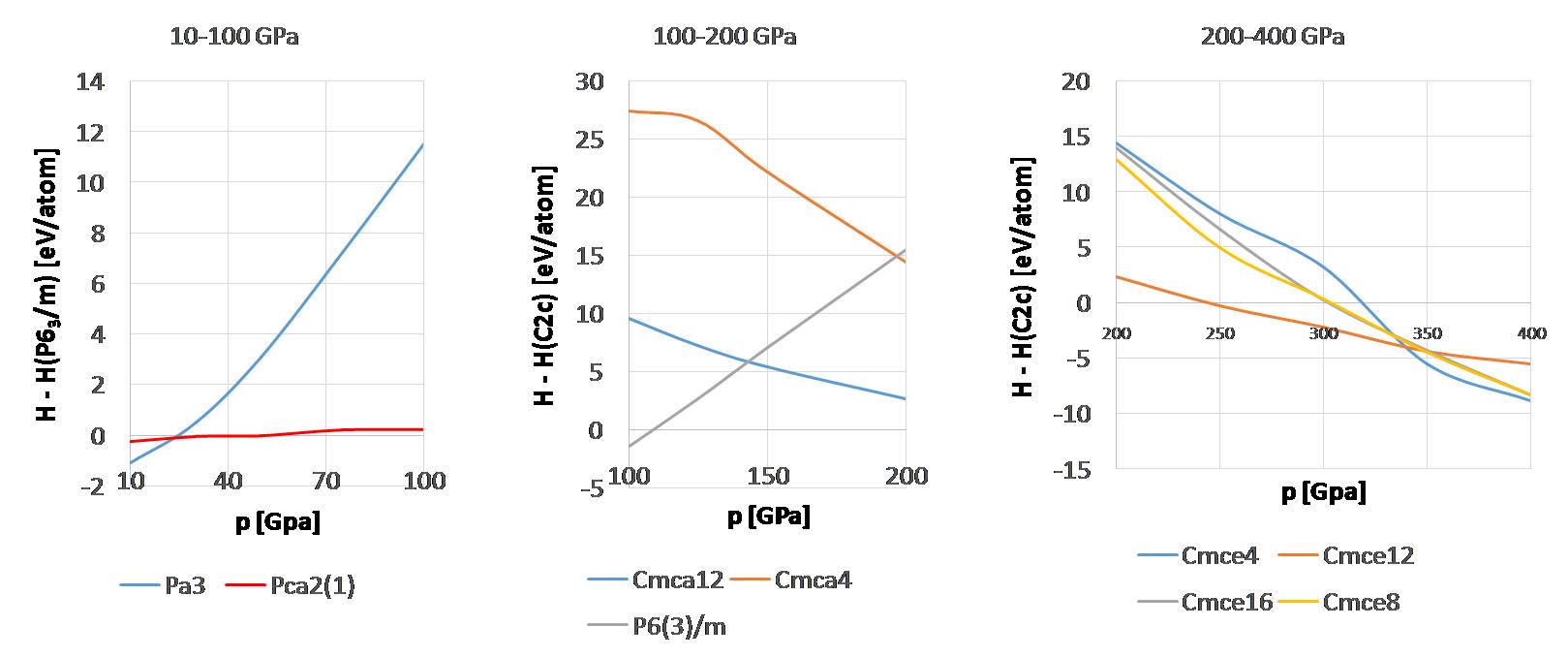


**Figure S3.** Comparison among the enthalpies of various H phases in the pressure range 10-400 GPa. Horizontal axis: pressure, in GPa. Vertical axis: enthalpy difference (meV/atom) with respect to the reference structure. The three plots refer to different pressure ranges, and the reference structures were *P6_3_/m*, *C2c* and *C2c* for the pressure ranges 10-100, 100-200 and 200-400, respectively. The following structures were obtained from our calculations: *Pa3*, *Pca2_1_*, *C2c*, *Cmce16*, *Cmce12*, *Cmce8*, while the remaining ones are the most stable presented in ref. 11. As several *Cmce* structures were found, we adopted the convention of ref. 11 and labeled them according to the number of atoms in the unit cell.

# Section S3. Additional QTAIM and ELF results for H_2_CO_3_, H_4_CO_4_ and H_2_O.


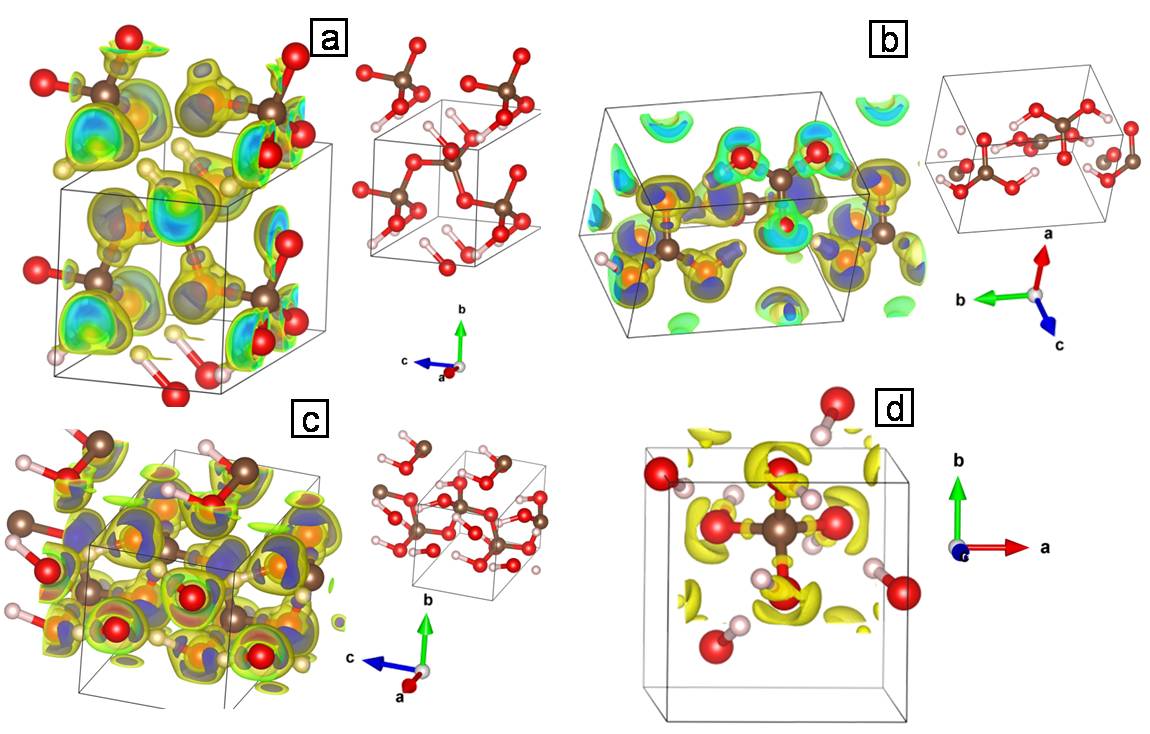


**Figure S4.** ELF isosurface plots of carbonic and orthocarbonic acids. (a) *Cmc2_1_*-H_2_CO_3_, 100 GPa (b) *Pnma*-H_2_CO_3_, 100 GPa (c) *Cmc2_1_*-H_2_CO_3_, 400 GPa (d) *I4_1_/a*-H_4_CO_4_, 400 GPa. (a-c) Isosurfaces values: 0.75 (yellow) and 0.85 (blue). The sections cutting isosurfaces are colored according to the ELF value from 0.5 (yellow) to 0.95 (blue), through green. Beside each picture, the structure is reported without isosurfaces, for sake of clarity. (d) Isosurfaces values is 0.85. All the O-H fragments acting as hydrogen bond donors towards the represented molecule are also shown. Note that in H_2_CO_3_ and H_4_CO_4_ the ELF around hydroxyl O atoms display the presence of two lone pairs, one of which is not involved in any strong hydrogen bond and has a larger isosurface, in agreement with the higher population of the associated basin (see Table 1 of the main text).


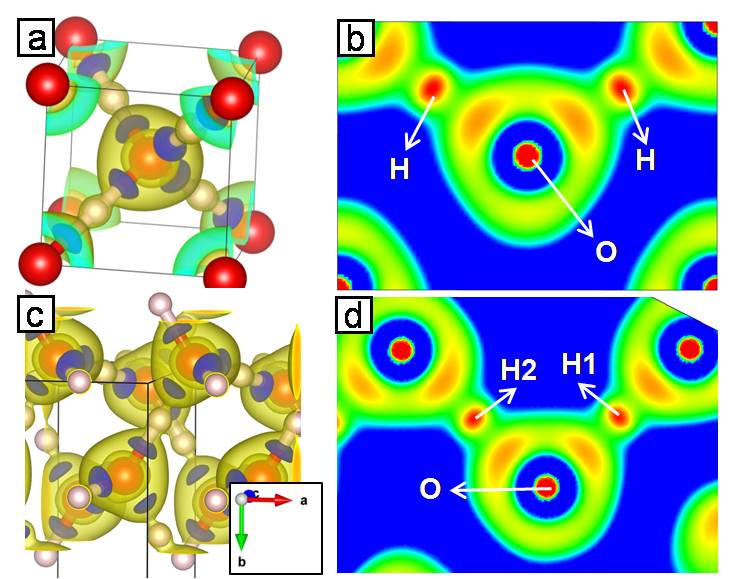


**Figure S5.** ELF distribution for ice X (100 GPa, a-b) and *Pbcm*-H_2_O (400 GPa, c-d). (a,c) isosurfaces ELF=0.75 (yellow) and 0.85 (blue). (b,d) ELF plot in the plane containing the atoms labeled in the picture (for *Pbcm*-H_2_O, the labels refer to Fig. 2d of the main text, while for ice X only one symmetry-independent atom of each type is present). Color scale as in Fig. 4a-b of the main text, *i.e.* from 0.5 (blue) to 0.95 (red).

**Table S2.** Bond lengths and properties at bond critical points of C-OH and C=O bonds of Pnma-H_2_CO_3_ at various pressures.

| P | bond | d_AB_ [Å] | ρ_BCP_ [e/a_0_^3^] | ellipticity |
| --- | --- | --- | --- | --- |
| 1 GPa | C=O | 1.258 | 0.382 | 0.141 |
|  | C-OH | 1.302 | 0.345 | 0.141 |
| 10 GPa | C=O | 1.263 | 0.377 | 0.135 |
|  | C-OH | 1.291 | 0.354 | 0.145 |
| 30 GPa | C=O | 1.265 | 0.375 | 0.128 |
|  | C-OH | 1.272 | 0.368 | 0.152 |
| 50 GPa | C=O | 1.265 | 0.374 | 0.120 |
|  | C-OH | 1.259 | 0.379 | 0.156 |
| 100 GPa | C=O | 1.260 | 0.378 | 0.105 |
|  | C-OH | 1.234 | 0.400 | 0.162 |

**Table S3.** Volume and population of all valence ELF basins of H_2_CO_3_, H_2_O and H_4_CO_4_ at selected pressures.

| basin | V[a_0_^3^] | n |  | basin | V[a_0_^3^] | n |
| --- | --- | --- | --- | --- | --- | --- |
| Cmc2_1_-H_2_CO_3_(100GPa) | | |  | Cmc2_1_-H_2_CO_3_(400GPa) | | |
| O1-C1 | 7.94 | 1.48 |  | O1-C1 | 6.5 | 1.53 |
| C1-O1 | 8.01 | 1.52 |  | C1-O1 | 7.12 | 1.58 |
| C1-O2 | 8.36 | 1.57 |  | C1-O2 | 7.49 | 1.7 |
| O2-H1 | 11.8 | 1.61 |  | O2-H1 | 8.61 | 1.66 |
| H1∙∙∙O2^a^ | 13.9 | 1.84 |  | H1∙∙∙O2 ^a^ | 8.33 | 1.63 |
| O1 l.p.^b^ | 19.3 | 2.40 |  | O1 l.p.^b^ | 12.83 | 2.34 |
| O2 l.p.^b^ | 21.2 | 2.55 |  | O2 l.p.^b^ | 14.2 | 2.56 |
| H1 | 3.27 | 0.28 |  | H1 | 2.42 | 0.3 |
| Pnma-H_2_CO_3_(100GPa) | | |  | I4_1_/a-H_4_CO_4_ (400GPa) | | |
| C1-O2 | 10.8 | 1.85 |  | C-O | 7.41 | 1.63 |
| C1-O1 | 12.6 | 2.04 |  | O-H | 8.51 | 1.67 |
| O1-H1 | 11.6 | 1.59 |  | H∙∙∙O ^a^ | 8.46 | 1.66 |
| H1∙∙∙O2 ^a^ | 15.9 | 1.98 |  | O l.p.^b^ | 13.9 | 2.56 |
| O2 l.p.^b^ | 16.1 | 1.82^b^ |  | H | 2.64 | 0.32 |
| O1 l.p.^b^ | 16.4 | 1.96 |  | Pbcm-H_2_O (400 GPa) | | |
| H1 | 4.96 | 0.38 |  | O-H2 | 10.1 | 1.87 |
|  | | |  | O-H1 | 10.1 | 1.8 |
|  |  |  |  | H1∙∙∙O ^a^ | 9.26 | 1.75 |
|  |  |  |  | H1 | 2.3 | 0.3 |
|  |  |  |  | H2 | 2.31 | 0.3 |

a) ELF basin acting as strong HBs acceptor (*vide infra*) b) l.p. refers to the ‘free’ lone pairs, *i.e.* those which are not acceptor of strong HBs (the latter defined as those having OHO angle and the H···O distance are greater than 130° and lower than 1.3 Å, respectively). For O atoms not acting as acceptor of any strong HB (hence formally having 2 ‘free’ lone pairs), we report the total lone pair population divided by two. c) note that, at 100 GPa, Pnma-H_2_CO_3_ displays 3 lone pairs on O2 (see Fig. 4 of the main text), one of which does not act as hydrogen bond acceptor.

# Section S4. C-H-O phase diagram and structural parameters of newly discovered crystal structures.


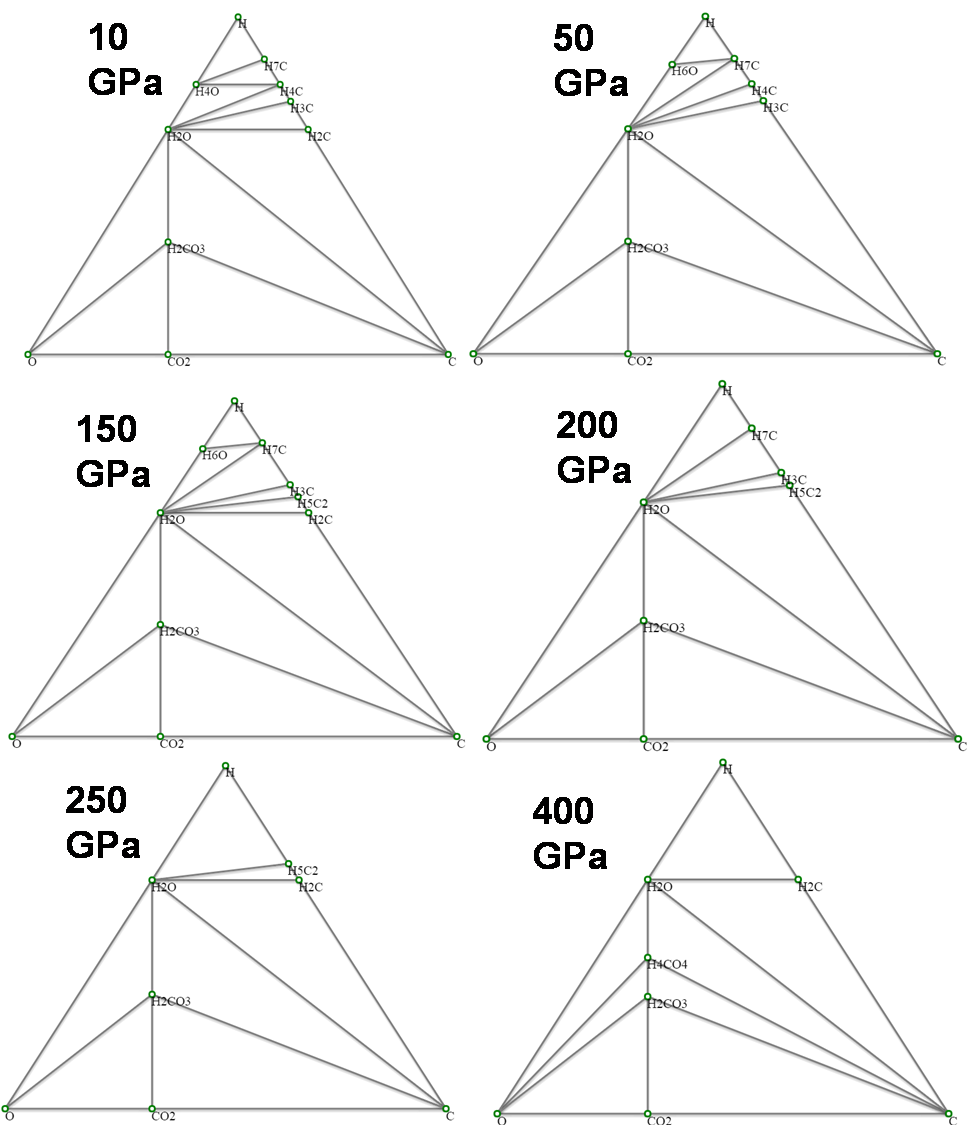


**Figure S6** Ternary phase diagrams of CHO at various pressures.

In the following, we report the structural parameters of newly discovered compounds/phases in the POSCAR format of VASP. Note that the latter can straightforwardly be converted into the CIF-format using the online utility at http://han.ess.sunysb.edu/poscar2cif/.

**H_2_CO_3_, Pnma, 1 GPa**

C H O

1.0

5.2199421877551089 0.0000000000000000 0.0000000000000000

0.0000000000000000 6.5624433542042153 0.0000000000000000

0.0000000000000000 0.0000000000000000 5.6267966869715114

4 8 12

Direct

0.6719504661967687 0.7500000000000002 0.0506272431062107

0.8280495338032317 0.2500000000000001 0.5506272431062107

0.3280495338032315 0.2500000000000001 0.9493727568937893

0.1719504661967686 0.7500000000000002 0.4493727568937893

0.1909469723457751 0.0409359437560084 0.4195113810090567

0.3090530276542250 0.5409359437560085 0.9195113810090567

0.8090530276542252 0.5409359437560085 0.5804886189909433

0.6909469723457752 0.0409359437560084 0.0804886189909433

0.8090530276542252 0.9590640562439918 0.5804886189909433

0.6909469723457752 0.4590640562439917 0.0804886189909433

0.1909469723457751 0.4590640562439917 0.4195113810090567

0.3090530276542250 0.9590640562439918 0.9195113810090567

0.2927538798081774 0.9104940395364787 0.3853374285490234

0.2072461201918227 0.4104940395364786 0.8853374285490234

0.7072461201918228 0.4104940395364786 0.6146625714509766

0.7927538798081776 0.9104940395364787 0.1146625714509766

0.9679989477450742 0.7500000000000002 0.5685097298323405

0.5320010522549261 0.2500000000000001 0.0685097298323405

0.0320010522549260 0.2500000000000001 0.4314902701676595

0.4679989477450742 0.7500000000000002 0.9314902701676595

0.7072461201918228 0.0895059604635216 0.6146625714509766

0.7927538798081776 0.5895059604635217 0.1146625714509766

0.2927538798081774 0.5895059604635217 0.3853374285490234

0.2072461201918227 0.0895059604635216 0.8853374285490234

**H_2_CO_3_, Cmc2_1_, 100 GPa**

1.0

6.9367104287639103 0.0000000000000000 0.0000000000000000

0.0000000000000000 4.0105216611412189 0.0000000000000000

0.0000000000000000 0.0000000000000000 3.6859945085537986

C H O

4 8 12

Direct

0.0000000000000000 0.8305731354751194 0.5697822800955896

0.0000000000000000 0.1694268645248806 0.0697822800955896

0.5000000000000000 0.3305731354751194 0.5697822800955896

0.5000000000000000 0.6694268645248806 0.0697822800955896

0.6661022829224110 0.0258312938459488 0.2002700364568581

0.3338977170775890 0.9741687061540513 0.7002700364568581

0.6661022829224110 0.9741687061540513 0.7002700364568581

0.3338977170775890 0.0258312938459488 0.2002700364568581

0.1661022829224110 0.5258312938459487 0.2002700364568581

0.8338977170775890 0.4741687061540514 0.7002700364568581

0.1661022829224110 0.4741687061540514 0.7002700364568581

0.8338977170775890 0.5258312938459487 0.2002700364568581

0.0000000000000000 0.1409214615767021 0.4383651462118590

0.1595439629443187 0.3328225145402731 0.9833891693966099

0.0000000000000000 0.8590785384232980 0.9383651462118590

0.1595439629443187 0.6671774854597270 0.4833891693966099

0.8404560370556813 0.3328225145402731 0.9833891693966099

0.8404560370556813 0.6671774854597270 0.4833891693966099

0.5000000000000000 0.6409214615767020 0.4383651462118590

0.6595439629443187 0.8328225145402730 0.9833891693966099

0.5000000000000000 0.3590785384232980 0.9383651462118590

0.6595439629443187 0.1671774854597270 0.4833891693966099

0.3404560370556813 0.8328225145402730 0.9833891693966099

0.3404560370556813 0.1671774854597270 0.4833891693966099

**H_2_CO_3_, Cmc2_1_, 400 GPa**

1.0

6.1632395801054374 0.0000000000000000 0.0000000000000000

0.0000000000000000 3.5283891486172143 0.0000000000000000

0.0000000000000000 0.0000000000000000 3.3507707692821023

C H O

4 8 12

Direct

0.5000000000000000 0.8143547688258561 0.7529532743419149

0.5000000000000000 0.1856452311741439 0.2529532743419149

0.0000000000000000 0.3143547688258561 0.7529532743419149

0.0000000000000000 0.6856452311741439 0.2529532743419149

0.7214125546556800 0.7592121038105235 0.3590750066275804

0.7214125546556800 0.2407878961894765 0.8590750066275804

0.2785874453443200 0.7592121038105235 0.3590750066275804

0.2785874453443200 0.2407878961894765 0.8590750066275804

0.2214125546556800 0.2592121038105235 0.3590750066275804

0.2214125546556800 0.7407878961894765 0.8590750066275804

0.7785874453443200 0.2592121038105235 0.3590750066275804

0.7785874453443200 0.7407878961894765 0.8590750066275804

0.5000000000000000 0.8394279571079970 0.1330794865715337

0.8340561042650594 0.1513676353901021 0.6370427772283912

0.1659438957349406 0.8486323646098979 0.1370427772283911

0.8340561042650594 0.8486323646098979 0.1370427772283911

0.1659438957349406 0.1513676353901021 0.6370427772283912

0.5000000000000000 0.1605720428920030 0.6330794865715337

0.0000000000000000 0.3394279571079970 0.1330794865715337

0.3340561042650594 0.6513676353901021 0.6370427772283912

0.6659438957349406 0.3486323646098979 0.1370427772283911

0.3340561042650594 0.3486323646098979 0.1370427772283911

0.6659438957349406 0.6513676353901021 0.6370427772283912

0.0000000000000000 0.6605720428920030 0.6330794865715337

**H_4_CO_4_, I4_1_/a, 400 GPa**

C H O

1.0

4.1221133653302253 0.0000000000000000 0.0000000000000000

0.0000000000000000 4.1221133653302253 0.0000000000000000

0.0000000000000000 0.0000000000000000 5.7751083387343689

4 16 16

Direct

0.5000000000000000 0.5000000000000000 0.5000000000000000

0.0000000000000000 0.5000000000000000 0.2500000000000000

0.0000000000000000 0.0000000000000000 0.0000000000000000

0.5000000000000000 0.0000000000000000 0.7500000000000000

0.8972318396817319 0.1658130024000457 0.7160207784677436

0.6027681603182681 0.3341869975999543 0.2160207784677435

0.3341869975999543 0.8972318396817319 0.4660207784677435

0.1658130024000457 0.1027681603182681 0.2839792215322564

0.1027681603182681 0.3341869975999543 0.5339792215322564

0.1658130024000457 0.6027681603182681 0.9660207784677436

0.3341869975999543 0.3972318396817318 0.7839792215322564

0.3972318396817318 0.1658130024000457 0.0339792215322564

0.3972318396817318 0.6658130024000457 0.2160207784677435

0.1027681603182682 0.8341869975999543 0.7160207784677435

0.8341869975999543 0.3972318396817318 0.9660207784677435

0.6658130024000457 0.6027681603182681 0.7839792215322564

0.6027681603182681 0.8341869975999543 0.0339792215322565

0.6658130024000457 0.1027681603182682 0.4660207784677435

0.8341869975999543 0.8972318396817318 0.2839792215322565

0.8972318396817318 0.6658130024000457 0.5339792215322564

0.4912758235723669 0.2520271099623244 0.3703353610487013

0.2479728900376756 0.9912758235723669 0.1296646389512987

0.7520271099623244 0.0087241764276331 0.1296646389512987

0.5087241764276331 0.7479728900376756 0.3703353610487013

0.5087241764276331 0.2479728900376756 0.8796646389512988

0.2520271099623244 0.0087241764276331 0.6203353610487012

0.2479728900376756 0.4912758235723669 0.1203353610487012

0.9912758235723669 0.2520271099623244 0.3796646389512987

0.9912758235723669 0.7520271099623244 0.8703353610487012

0.7479728900376756 0.4912758235723669 0.6296646389512988

0.2520271099623244 0.5087241764276331 0.6296646389512988

0.0087241764276331 0.2479728900376756 0.8703353610487012

0.0087241764276331 0.7479728900376756 0.3796646389512988

0.7520271099623244 0.5087241764276331 0.1203353610487012

0.7479728900376756 0.9912758235723669 0.6203353610487012

0.4912758235723669 0.7520271099623244 0.8796646389512988

**2CH_4_:3H_2_, P-1, 200GPa**

1.0

2.7281174665945525 0.0000000000000000 0.0000000000000000

-1.3061257128551764 3.7007574853540435 0.0000000000000000

-1.3364707664592035 -1.6081166169245364 3.3516711069940555

C H

2 14

Direct

0.9696212180482647 0.2104062343985097 0.3054487717063036

0.0303787819517353 0.7895937656014903 0.6945512282936964

0.7400479217401833 0.1866614464736465 0.6568285374428753

0.3751431090985300 0.7381039303978477 0.6867178632319313

0.6422993120209820 0.5074040565116463 0.0602479410497200

0.2522252945560227 0.7673131268355839 0.1534858344378504

0.6248568909014700 0.2618960696021523 0.3132821367680687

0.2599520782598167 0.8133385535263535 0.3431714625571247

0.7477747054439773 0.2326868731644161 0.8465141655621495

0.1958883420144469 0.0752653924209551 0.9028597937864858

0.7591983163370242 0.7630505572432000 0.4218719488986086

0.8041116579855532 0.9247346075790449 0.0971402062135142

0.2408016836629758 0.2369494427568000 0.5781280511013913

0.7866381597408197 0.5789087005830201 0.7612599498865785

0.2133618402591803 0.4210912994169799 0.2387400501134215

0.3577006879790180 0.4925959434883537 0.9397520589502800

**O, P6_3_/mmc, 400 GPa**

1.0

1.8778888884838376 0.0000000000000000 0.0000000000000000

-0.9389444442419188 1.6262994829115260 0.0000000000000000

0.0000000000000000 0.0000000000000000 5.4410668904939010

O

4

Direct

0.6666666666666666 0.3333333333333333 0.3539919641276312

0.6666666666666667 0.3333333333333334 0.1460080358723688

0.3333333333333333 0.6666666666666666 0.8539919641276312

0.3333333333333334 0.6666666666666667 0.6460080358723688

**2H_2_:H_2_O, I4_1_/a, 100 GPa**

1.0

3.7588261430740926 0.0000000000000000 0.0000000000000000

0.0000000000000000 3.7588261430740926 0.0000000000000000

0.0000000000000000 0.0000000000000000 5.2662285896243040

H O

24 4

Direct

0.0000000000000000 0.2500000000000000 0.1250000000000000

0.2500000000000000 0.5000000000000000 0.3750000000000000

0.1878205722609851 0.5633966279445933 0.8507667405047962

0.1878205722609851 0.0633966279445932 0.3992332594952038

0.7500000000000000 0.5000000000000000 0.3750000000000000

0.0000000000000000 0.7500000000000000 0.1250000000000000

0.5633966279445933 0.3121794277390149 0.1007667405047963

0.3121794277390149 0.4366033720554067 0.8992332594952038

0.0633966279445932 0.3121794277390149 0.6492332594952038

0.3121794277390149 0.9366033720554067 0.3507667405047963

0.9366033720554067 0.1878205722609851 0.6007667405047963

0.4366033720554067 0.1878205722609851 0.1492332594952038

0.5000000000000000 0.7500000000000000 0.6250000000000000

0.7500000000000000 0.0000000000000000 0.8750000000000000

0.6878205722609851 0.0633966279445932 0.3507667405047963

0.6878205722609851 0.5633966279445932 0.8992332594952038

0.2500000000000000 0.0000000000000000 0.8750000000000000

0.5000000000000000 0.2500000000000000 0.6250000000000000

0.0633966279445932 0.8121794277390149 0.6007667405047963

0.8121794277390149 0.9366033720554067 0.3992332594952037

0.5633966279445932 0.8121794277390149 0.1492332594952037

0.8121794277390149 0.4366033720554068 0.8507667405047963

0.4366033720554068 0.6878205722609851 0.1007667405047963

0.9366033720554067 0.6878205722609851 0.6492332594952038

0.0000000000000000 0.5000000000000000 0.2500000000000000

0.0000000000000000 0.0000000000000000 0.0000000000000000

0.5000000000000000 0.0000000000000000 0.7500000000000000

0.5000000000000000 0.5000000000000000 0.5000000000000000

**C_2_H_6_, P2_1_/c, 200 GPa**

1.0

2.6709005766214706 0.0000000000000000 0.0000000000000000

0.0000000000000000 3.8264235754695224 0.0000000000000000

-2.6714028235280485 0.0000000000000000 3.8586430573609736

C H

4 12

Direct

0.6910025346832875 0.6336616579229810 0.1219426604321964

0.3089974653167125 0.1336616579229810 0.3780573395678036

0.6910025346832875 0.8663383420770190 0.6219426604321964

0.3089974653167125 0.3663383420770190 0.8780573395678036

0.4846448521368403 0.6475781609315716 0.5599397389311911

0.5153551478631597 0.1475781609315716 0.9400602610688089

0.4846448521368403 0.8524218390684284 0.0599397389311911

0.5153551478631597 0.3524218390684284 0.4400602610688089

0.7924335782920197 0.9368755368743318 0.8653281461651265

0.2075664217079803 0.4368755368743318 0.6346718538348735

0.7924335782920197 0.5631244631256682 0.3653281461651265

0.2075664217079803 0.0631244631256682 0.1346718538348735

0.9022306378937971 0.3690279073726891 0.8531872118275596

0.0977693621062029 0.8690279073726891 0.6468127881724404

0.9022306378937971 0.1309720926273109 0.3531872118275596

0.0977693621062029 0.6309720926273109 0.1468127881724404

# Section S5. H_2_CO_3_ formation pressure calculated with different basis sets and DFT functionals.

In the following we report the values of the formation pressure of H_2_CO_3_ obtained with various methods. Besides the plane-waves, we present the results obtained from calculations in which the crystal orbitals are described as a linear combination atom-centered functions whose radial part takes the form of Gaussian functions. These calculations were performed with the CRYSTAL14 code^[[14]](#endnote-14)^. Details concerning the implementation of the various exchange-correlation functionals and the corresponding references can be found in the manual^[[15]](#endnote-15)^. The basis set is the same as described in the ‘Methods’ section of the main text, except that we did not apply any contraction factor to the radial part of the basis functions. For some DFT functionals, we added the empirical dispersion correction term developed by Grimme^[[16]](#endnote-16)^ (indicated as ‘+D’ in the table).

**Table S4** Formation pressure of H_2_CO_3_ obtained from different DFT-Hamiltonian/basis sets.

| functional | basis set | formation pressure (GPa) |
| --- | --- | --- |
| optB88-vdw^a^ | plane waves^a^ | 0.95 |
| B3LYP+D^b^ | atom-centered | 1.05 |
| B97H+D^c^ | atom-centered | 0.2 |
| PBE+D | atom-centered | 0.0^d^ |

a) Details in the ‘methods’ section of the main text

b) The dispersion parameters d and s6 we adopted were different from the original one defined in ref. 15, and were taken from ref. ^[[17]](#endnote-17)^.

c) This is the hybrid version of the B97 functional, for which the dispersion parameters d and s6 were not reported in ref. 15. Therefore, we adopted the values relative to the B97 functional

d) H_2_CO_3_ already stable at ambient pressure.

These results refer to the enthalpy contributions without the zero-point vibrational energy correction. The ranges reported in the main text (*i.e.* the “realistic estimation for the formation pressure of carbonic acid”) were obtained by averaging the values reported in Table S4, adding the standard deviation of the average (±0.5 GPa) and adding the increase in the formation pressure (see Fig. 1c-d of the main text) determined from opt88-vdw plane wave calculations. We assume that the increase in the formation pressure caused by phonons contribution does not depend significantly on the adopted exchange-correlation functional and/or the basis set.

# Section S6. Phonon dispersion curves of newly discovered compounds.

In this section, we report the phonon dispersion curves for the newly discovered compounds obtained by the finite displacement approach as implemented in the PHONPY code^[[18]](#endnote-18)^, with the required force calculations done using VASP. Prior to the generation of ‘displaced structures’ (*i.e.* the structures in which atoms are displaced from their equilibrium positions in order to implement the finite displacement approach), the geometry was optimized (*i.e.* the enthalpy was minimized with respect to cell shape and volume and atomic positions) until the forces on each atom were lower than 1*10^-8^ eV/Å. Moreover, to minimize the possible bias due to Pulay stress, we reiterated the geometry optimization until the forces in the first optimization step were lower than 3*10^-6^ eV/Å. The self-consistent field (SCF) procedure of each optimization step was considered converged when the energy difference between two successive SCF cycles was lower than 1*10^-8^ eV. The same convergence threshold was applied to the SCF procedure in the subsequent single-point calculations on the ‘displaced structures’. The same reciprocal space sampling scheme and plane-wave energy cutoff as described in the Methods section were adopted, except for the calculations at 0, 1 and 2 GPa (used to obtain phonon contribution to the free energy of formation of carbonic acid), for which we used a k-grid resolution of 0.7 Å^-1^ and a plane-wave energy cutoff of 750 eV. This is because for ice-XV, which is the most stable phase of water (reactant) at the H_2_CO_3_ formation pressure (see Supplementary Section 2), a very large supercell (3x3x3, 810 atoms) was to be adopted in order to avoid artificial imaginary frequencies. Performing such calculations with 850 eV energy cutoff and 0.40 Å^-1^ reciprocal space sampling was too computationally demanding. The adopted settings can be anyway considered reasonably accurate in light of the facts that: 1) a dense reciprocal space sampling is not necessary for molecular crystals, as long-distance orbital interactions are expected to be of minor importance 2) the adopted plane-wave energy cutoff is anyway higher than the highest among the VASP default values assigned to the adopted pseudopotentials (700 eV). Note that the phonon-independent enthalpy contribution to the free energy of formation was anyway obtained with the more accurate settings described in the Methods section of the main text.


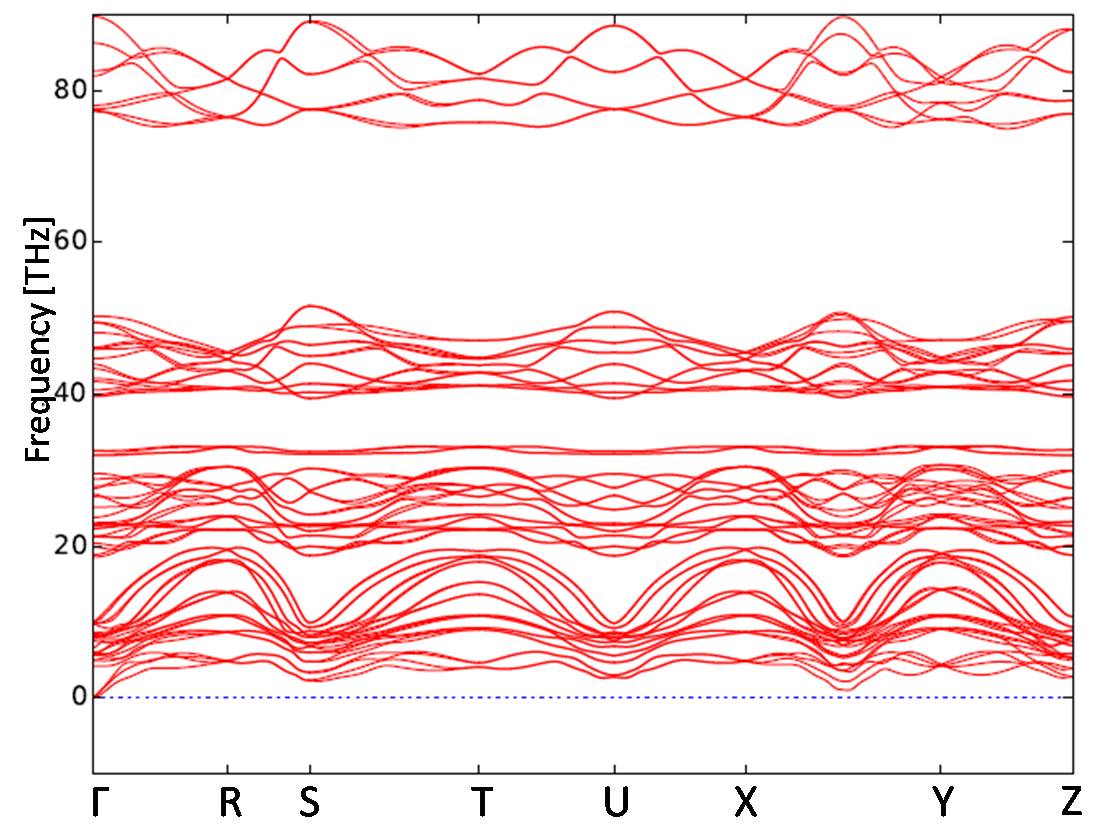


**Figure S7** Phonon dispersion curves of *Pnma*-H_2_CO_3_ at 2 GPa (2x3x2 supercell, 288 atoms)


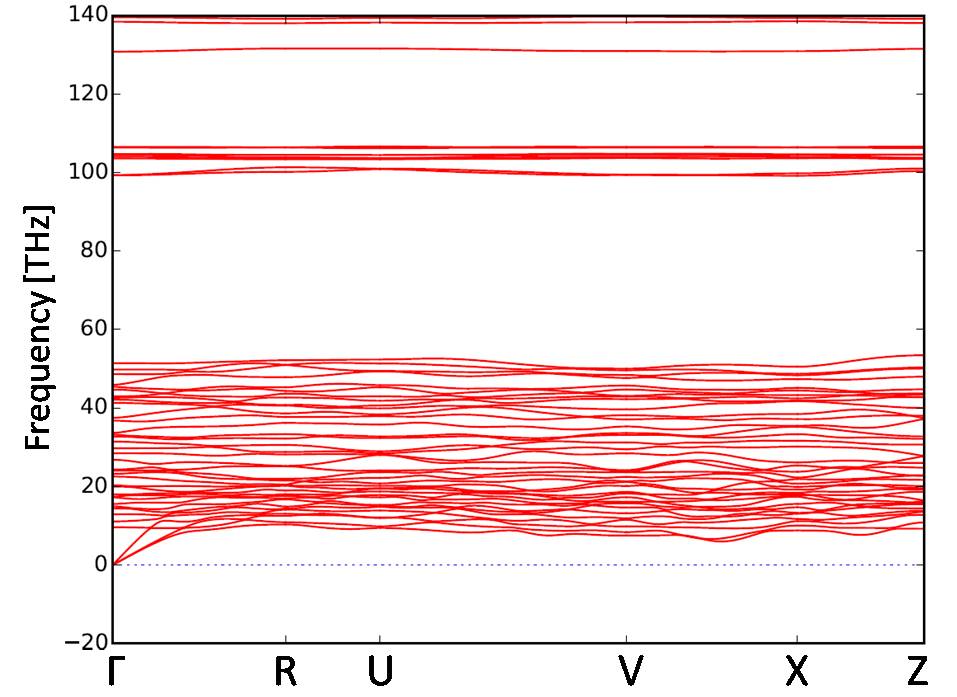


**Figure S8** Phonon dispersion curves of 2CH_4_:3H_2_ (*P-1* space group) at 100 GPa (2x2x3 supercell, 192 atoms)


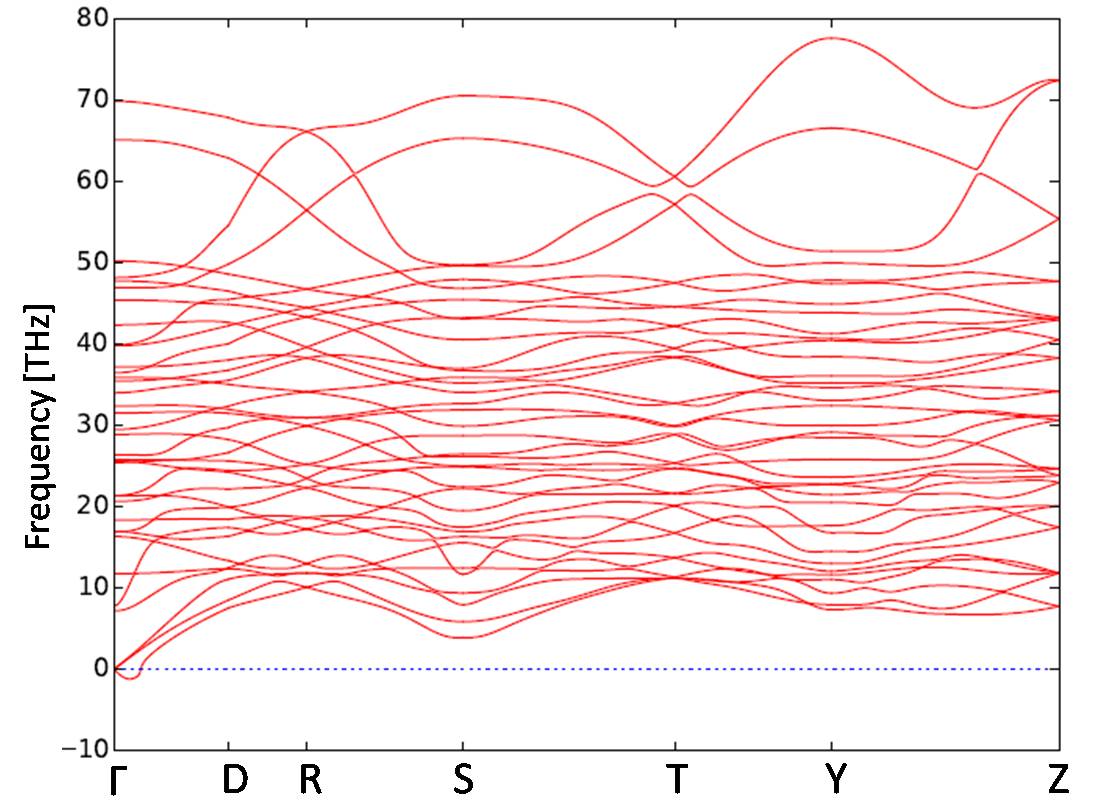


**Figure S9** Phonon dispersion curves of *Cmc2_1_*-H_2_CO_3_ at 100 GPa (3x3x3 supercell, 324 atoms). Note that one of the acoustic modes has a tiny imaginary part near the Γ-point; this is a numerical artifact.


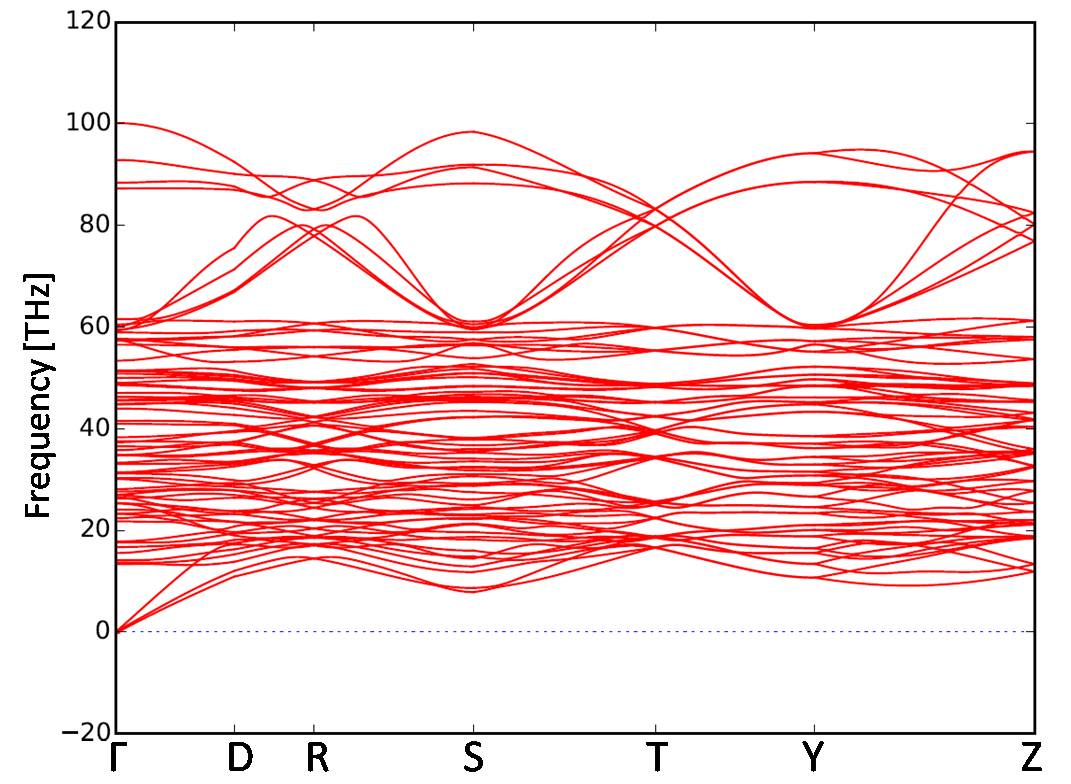


**Figure S10** Phonon dispersion curves of *Cmc2_1_*-H_2_CO_3_ at 400 GPa (2x2x3 supercell, 288 atoms)


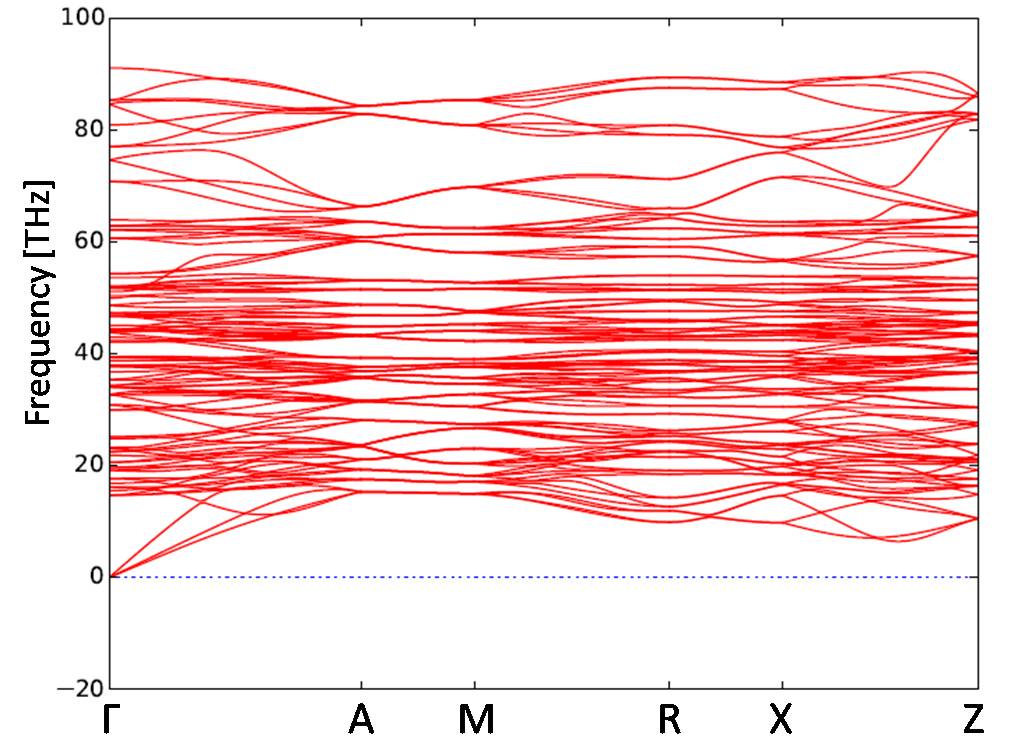


**Figure S11** Phonon dispersion curves of *I4_1_/a*-H_4_CO_4_ at 400 GPa (2x2x2 supercell, 288 atoms)


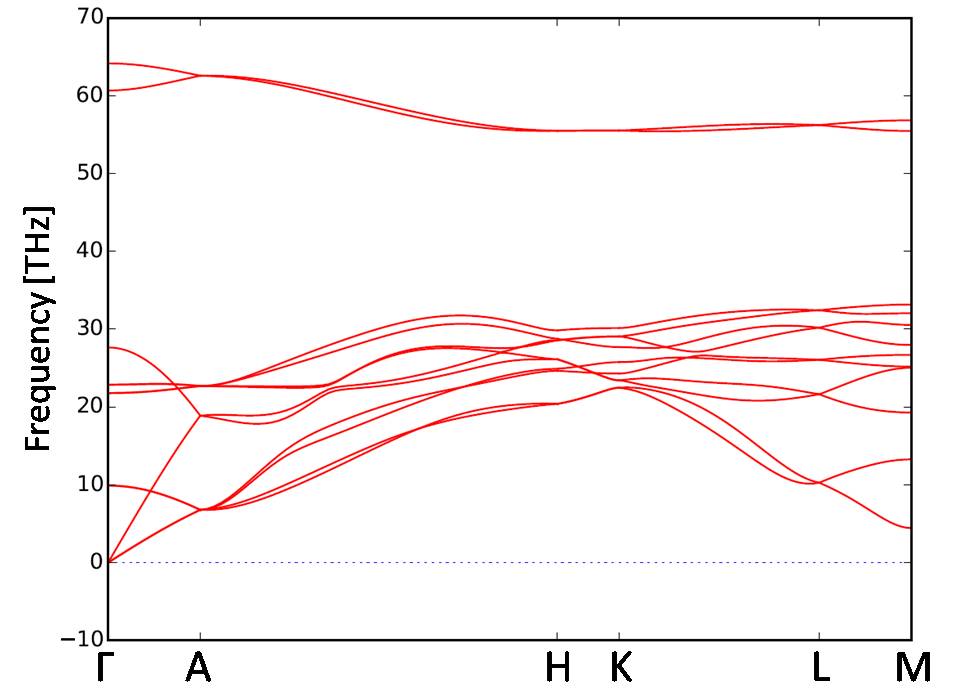


**Figure S12** Phonon dispersion curves of *P6_3_/mmc*-O at 400 GPa (5x5x3 supercell, 300 atoms)

# Section S7. Additional representations of 2CH_4_:3H_2_ crystal structure


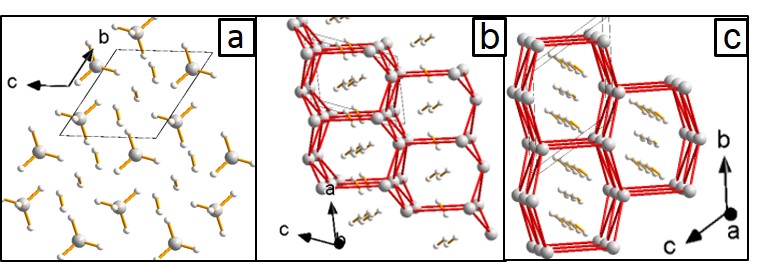


**Figure S13.** Crystal structure of 2CH_4_:3H_2_. In order to show the topology of the host framework, the structure is shown in (b) and (c) in different orientation, without the hydrogen atoms of methane and with red lines joining nearest neighbor carbon atoms.

# Section S8. Structure and bonding of a polymorph of H_4_CO_4_ .

**
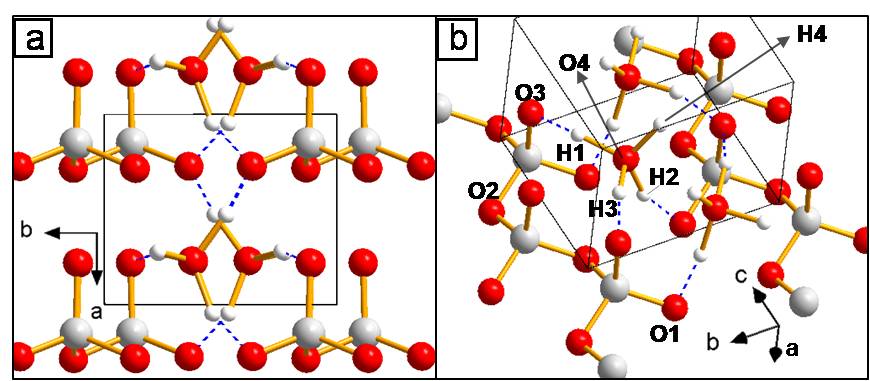
**

**Figure S14** Crystal structure of H_4_CO_4_, space group Pc. In (b), we show half of the atoms shown in (a) with a different orientation, along with the atom numbering used in Table S5. Only symmetry-independent atoms are labeled. This polymorph of H_4_CO_4_ is less stable than orthocarbonic acid (space group I4_1_/a) above 310 GPa but more stable than the reactant mixture H_2_CO_3_+H_2_O above 395 GPa.


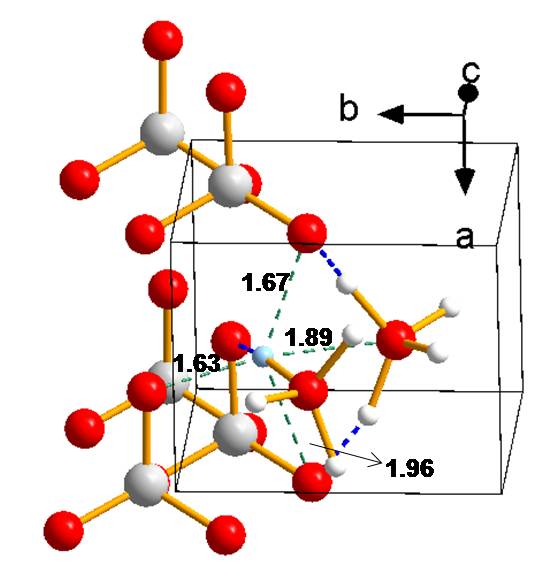


**Figure S15** Hydrogen bonds in Pc-H_4_CO_4_. The coordination sphere of H1 atom (see Fig. S14), colored in light blue, is shown. The remaining H atoms have a veery similar coordination (not shown). The short H···O contacts shown reported in Fig. S14 are represented by dotted, thick, blue lines. All other H···O contacts within 2.0 Å are indicated by dotted, thin, green lines (lengths reported in Å). The OHO angles relative to O-H-O bonds (displayed here and in Fig. S14 as thick solid + blue dotted lines) for the 4 symmetry-independed H atoms are: 158.5° (H1), 157.4° (H2), 159.0° (H3) and 159.6° (H4).

**Table S5.** ELF basins properties (volume and electron population) of *Pc*-H_4_CO_4_ and their variation along the homodesmic reaction H_2_O+H_2_CO_3_🡪 (Pc) H_4_CO_4_ at 400 GPa.

| **V [a_0_^3^]**^a^ | **N [e]**^b^ | **label** |  | **V [a_0_^3^]** | **N [e]** | **label** |
| --- | --- | --- | --- | --- | --- | --- |
| C-O | | |  | O-H | | |
| 6.86 | 1.58 | C-O2 |  | 9.65 | 1.82 | O4-H1 |
| 6.88 | 1.58 | O2-C |  | 9.16 | 1.80 | O4-H4 |
| 7.25 | 1.62 | C-O3 |  | 8.48 | 1.68 | O4-H3 |
| 7.14 | 1.65 | C-O1 |  | 10.13 | 1.89 | O4-H2 |
| C-O total variation^c^ | | |  | H···O | | |
| **-0.48** | **-0.07** |  |  | 8.84 | 1.70 | H1···O3 |
| free lone pairs | | |  | 8.05 | 1.55 | H4···O2 |
| 13.73 | 2.53 | O3 |  | 9.04 | 1.73 | H3···O3 |
| 16.44 | 2.87 | O1 |  | 7.76 | 1.50 | H2···O2 |
| 12.96 | 2.32 | O2 (2) |  | H | | |
| free lone pairs tot. var.^c^ | | |  | 2.56 | 0.31 | H1 |
| **2.10** | **0.23** |  |  | 2.52 | 0.31 | H4 |
|  | | |  | 2.47 | 0.31 | H3 |
|  |  |  |  | 2.38 | 0.30 | H2 |
|  |  |  |  | O-H···O tot. var. ^c,d^ | | |
|  |  |  |  | **-1.83** | **-0.15** |  |

a) volume in a.u. b) electron population in a.u. c) total variation along the homodesmic reaction H_2_O+H_2_CO_3_🡪 (Pc) H_4_CO_4_ d) the 4 symmetry-independent O-H···O interactions of H_4_CO_4_ (see Fig. S14) are compared to those of water (two) and carbonic acid (remaining two). The volume changes are +2.05 a.u. and -3.87 a.u. with respect to carbonic acid and water, respectively. The population changes are +0.30 e and -0.45 e with respect to carbonic acid and water, respectively.

# Section S9. Comparison of calculated IR and Raman spectra of H_2_CO_3_ with previously reported experimental measurements.

The IR and Raman spectra of carbonic acid formed by mild compression of H_2_O:CO_2_ mixture were reported by Wang *et al.*^[[19]](#endnote-19)^. Here we compare them with the spectra calculated from our crystal structure (Pnma space group) whose geometry was optimized at 3.5 GPa. We exploited the code CRYSTAL14^13^ to obtain the spectra by means of the coupled perturbed Kohn-Sham approach^[[20]](#endnote-20),^^[[21]](#endnote-21),^^[[22]](#endnote-22)^(within the harmonic approximation). The adopted basis set was the same as described in the “method” section of the main text.


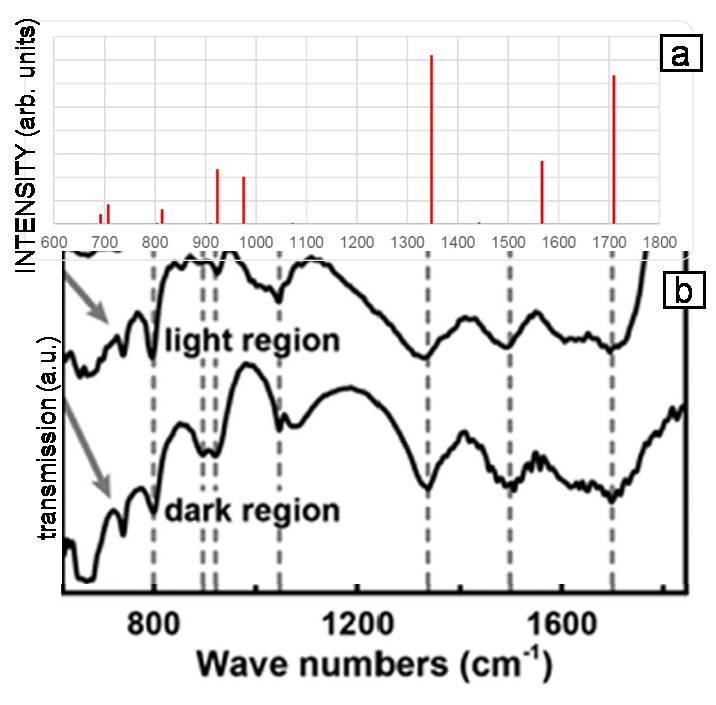


**Figure S16.** Calculated (a) and experimental (b; from ref. 18, ambient temperature, 3.5 GPa) IR spectra. The spectrum labeled as “light region” corresponds to the one Wang *et al.*^18^ identify as containing crystalline H_2_CO_3_. The grey arrows indicate the peaks assigned to unreacted CO_2_.


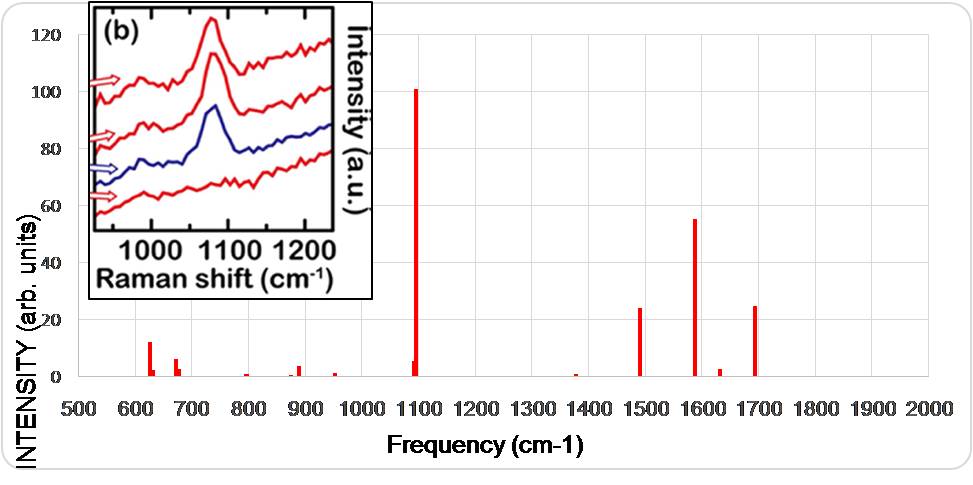


**Figure S17**. Calculated Raman spectrum of Pnma-H_2_CO_3_. The experimental Raman spectrum taken from ref. 18 is reported in the upper-left inset; the various curves correspond to various parts of the sample, the upmost three being those where carbonic acid is present.

The experimental and calculated IR spectra show an overall good agreement. Note that the experimental spectra displays two peaks around 900 cm^-1^, while only one is present in the calculated spectra. However, other reported IR measurements (*e.g.* ref. ^[[23]](#endnote-23)^), including that used as reference by Wang *et al.*, display only one peak at such frequency. Hence, our calculations leads to the same number of peaks as the experimental spectra. Two of them (the ones at 976 and 1567 cm^-1^ in the calculated spectra) show some difference between experiment and theory - however, differences of this magnitude are occasionally observed when comparing harmonic DFT frequencies with experimental ones^[[24]](#endnote-24)^.

Concerning the Raman spectra, Wang *et al.* observed a strong peak at 1073 cm^-1^ and two, weaker ones at 689 and 635 cm^-1^. All of them are present in our spectrum. Again, the frequency discrepancy between calculated and observed peaks are well inside the range of typical errors made by harmonic DFT calculations^23^. The further three peaks in the 1500-1800 cm^-1^ region are in agreement with previously reported Raman spectra^22^.

Two further points need to be discussed:

**The decomposition pressure**. Wang *et al.* performed two sets of experiments to measure the pressure at which H_2_CO_3_ would form/decompose. In the first one, the whole sample was resistively heated to 180° C at various pressures, and H_2_CO_3_ was observed only when the applied pressure was greater or equal than 2.4 GPa. In these experiments, the sample was liquid, and H_2_CO_3_ formed as aqueous solution. Therefore, the measured formation pressure cannot be directly compared with our results, which refer to solid state. In the other set of experiments, H_2_CO_3_ was formed by leaser heating the compressed gasket. Specifically, carbonic acid was observed to form in solid state at the periphery of the laser-heated spot. The decomposition pressure was measured by letting the sample cooling down at room temperature and then decompressing it. The same decomposition pressure of 2.4 GPa was observed, thus deviating from our estimated range of 0.75-1.75 GPa for carbonic acid formation at 300 K (see main text). Such a difference should come as no surprise: while our calculations refer to pure, solid H_2_CO_3_, the experiments were performed on the H_2_O/CO_2_/H_2_CO_3_ ternary system, part of which was likely to be in liquid state (as also stated by the authors: “the decomposition to H_2_O and CO_2_ is exactly the behavior that is conventionally expected for solid H_2_CO_3_ in contact with liquid water”). Therefore, the experimentally observed decomposition pressure is influenced by a number of factors other than the thermodynamics of H_2_CO_3_ 🡪 CO_2_ + H_2_O reaction^[[25]](#endnote-25)^. Just to name a few possible factors which may influence the decomposition pressure of carbonic acid in the sample: the presence of (liquid) water^[[26]](#endnote-26)^, pressure- and temperature-induced shifts in the equilibrium H_2_CO_3_ + H_2_O ↔ HCO_3_^-^ + H_3_O^+^ (also detected by Wang *et al.*^18^), which, in turn, influence the melting temperature/pressure of the various species in the sample^[[27]](#endnote-27)^.

**Pressure changes in the sample upon carbonic acid formation/decomposition**. Wang *et al.*^18^ observed a pressure increase (drop) when carbonic acid forms (decomposes). As a consequence, they hypothesize that H_2_CO_3_ should have a volume larger than the H_2_O+CO_2_ mixture. On the contrary, we found that, throughout the investigated pressure range (0-400 GPa), H_2_CO_3_ becomes stable because of the volume reduction over the reactant mixture, which favors the pV term of the free energy. This holds true for all tested computational approaches (described in Supplementary Section 5). Our results agree with what one would expect for a pressure-stabilized compound: an increase in volume upon its formation would lead to a pressure-induced decomposition, but Wang *et al.* observed no decomposition of H_2_CO_3_ up to the highest pressure reached during the experiment, namely 25 GPa. We note that the volume increase was not observed in the resistive heating experiment (or at least it was not mentioned in the paper), where the whole sample was liquid. Therefore, we hypothesize that the observed volume changes could be caused by pressure-induced or temperature-induced shifts in solid-liquid equilibriums.

# REFERENCES

1. Klimeš, J., Bowler, D. & Michaelides, A. Van der Waals density functionals applied to solids. *Phys. Rev. B* 83, (2011). [↑](#endnote-ref-2)
2. Qian, G., Lyakhov, A., Zhu, Q., Oganov, A. & Dong, X. Novel Hydrogen Hydrate Structures under Pressure. *Sci. Rep.* 4, (2014). [↑](#endnote-ref-3)
3. Caracas, R. Dynamical instabilities of ice X. *Phys. Rev. let.*, 101, 085502 (2008). [↑](#endnote-ref-4)
4. Benoit, M., Bernasconi, M., Focher, P. & Parrinello, M. New High-Pressure Phase of Ice, *Phys. Rev. Lett.* 76, 2934-2936 (1996). [↑](#endnote-ref-5)
5. Salzmann, C. G., Radaelli, P. G., Mayer, E., & Finney, J. L. Ice XV: a new thermodynamically stable phase of ice. *Phys. Rev. Lett.*, *103*, 105701 (2009). [↑](#endnote-ref-6)
6. Ma, Y., Oganov, A. & Glass, C. Structure of the metallic ζ-phase of oxygen and isosymmetric nature of the ε-ζ phase transition: Ab initio simulations. *Phys. Rev. B* 76, (2007). [↑](#endnote-ref-7)
7. Note that, within the GGA approximation, the *Cmcm* structure of oxygen results more stable than the experimentally determined ε phase, whose space group is *C2/m* (Fujihisa H. *et al.*, O8 Cluster Structure of the Epsilon Phase of Solid Oxygen *Phys. Rev. Lett.* 97, 085503 (2006) ). In our calculations, the stability ranges and the phase diagram were determined considering the most stable allotropes (including the *C2/m* ε phase just mentioned) at each pressure. [↑](#footnote-ref-2)
8. Lu, C., Miao, M. & Ma, Y. Structural Evolution of Carbon Dioxide under High Pressure. *J. Am. Chem. Soc.* 135, 14167-14171 (2013). [↑](#endnote-ref-8)
9. Yoo, C. S. *et al.* Crystal structure of pseudo-six-fold carbon dioxide phase II at high pressures and temperatures. *Phys. Rev. B*, 65, 104103 (2002). [↑](#endnote-ref-9)
10. Dalladay-Simpson, P., Howie, R. T., & Gregoryanz, E. Evidence for a new phase of dense hydrogen above 325 gigapascals. *Nature*, 529, 63-67 (2016). [↑](#endnote-ref-10)
11. Silvera, I. F. The solid molecular hydrogens in the condensed phase: Fundamentals and static properties. *Rev. Mod. Phys.* 52, 393-452 (1980). [↑](#endnote-ref-11)
12. Pickard, C. & Needs, R. Structure of phase III of solid hydrogen. *Nat. Phys.* 3, 473-476 (2007). [↑](#endnote-ref-12)
13. Gao, G. *et al*. Dissociation of methane under high pressure. *J. Chem. Phys.* 133, 144508 (2010). [↑](#endnote-ref-13)
14. Dovesi, R. et al. CRYSTAL14 : A program for the ab initio investigation of crystalline solids. *Int. J. Quantum Chem.* 114, 1287-1317 (2014). [↑](#endnote-ref-14)
15. http://www.crystal.unito.it/Manuals/crystal14.pdf [↑](#endnote-ref-15)
16. Grimme, S. Semiempirical GGA‐type density functional constructed with a long‐range dispersion correction. *J. Comput. Chem.*, *27*(15), 1787-1799 (2006). [↑](#endnote-ref-16)
17. Civalleri, B., Zicovich-Wilson, C. M., Valenzano, L., & Ugliengo, P.. B3LYP augmented with an empirical dispersion term (B3LYP-D*) as applied to molecular crystals. *CrystEngComm*, *10*(4), 405-410 (2008). [↑](#endnote-ref-17)
18. Togo, A. & Tanaka, I. First principles phonon calculations in materials science, *Scr. Mater.*, 108, 1-5 (2015). [↑](#endnote-ref-18)
19. Wang, H., Zeuschner, J., Eremets, M., Troyan, I., & Willams, J. Stable solid and aqueous H_2_CO_3_ from CO_2_ and H_2_O at high pressure and high temperature. *Sci. Rep.*, 6: 19902 (2016). [↑](#endnote-ref-19)
20. Pascale, F. *et al.* The calculation of the vibrational frequencies of crystalline compounds and its implementation in the CRYSTAL code. *J. Comput. Chem.* 25, 888-897 (2004). [↑](#endnote-ref-20)
21. Maschio, L., Kirtman, B., Orlando, R., & Rèrat, M. Ab initio analytical infrared intensities for periodic systems through a coupled perturbed Hartree-Fock/Kohn-Sham method. *J. Chem.. Phys.*, *137*, 204113(2012). [↑](#endnote-ref-21)
22. Maschio, L., Kirtman, B., Rérat, M., Orlando, R., & Dovesi, R. Ab initio analytical Raman intensities for periodic systems through a coupled perturbed Hartree-Fock/Kohn-Sham method in an atomic orbital basis. I. Theory *J. Chem.. Phys.* 139, 164101 (2013). [↑](#endnote-ref-22)
23. Kohl, I *et al.* Raman Spectroscopic Study of the Phase Transition of Amorphous to Crystalline β‐Carbonic Acid. *Angew. Chem. Int. Ed.* 48 2690-2694 (2009). [↑](#endnote-ref-23)
24. Malik, M., Wysokiński, R., Zierkiewicz, W., Helios, K., & Michalska, D. Raman and infrared spectroscopy, DFT calculations, and vibrational assignment of the anticancer agent picoplatin: Performance of long-range corrected/hybrid functionals for a platinum (II) complex. *J. Phys. Chem. A*, *118*, 6922-6934 (2014). [↑](#endnote-ref-24)
25. Hongbo Wang, Janek Zeuschner. *Personal communication.* [↑](#endnote-ref-25)
26. Tautermann, C. et al. Towards the Experimental Decomposition Rate of Carbonic Acid (H_2_CO_3_) in Aqueous Solution. *Chem.-Eur. J.* 8, 66-73 (2002). [↑](#endnote-ref-26)
27. Journaux, B., Daniel, I., Caracas, R., Montagnac, G., & Cardon, H. Influence of NaCl on ice VI and ice VII melting curves up to 6GPa, implications for large icy moons. *Icarus*, 226, 355-363 (2013). [↑](#endnote-ref-27)
